# A transcriptomic map of EGFR-induced epithelial-to-mesenchymal transition identifies prognostic and therapeutic targets for head and neck cancer

**DOI:** 10.1186/s12943-022-01646-1

**Published:** 2022-09-08

**Authors:** Henrik Schinke, Enxian Shi, Zhongyang Lin, Tanja Quadt, Gisela Kranz, Jiefu Zhou, Hongxia Wang, Julia Hess, Steffen Heuer, Claus Belka, Horst Zitzelsberger, Udo Schumacher, Sandra Genduso, Kristoffer Riecken, Yujing Gao, Zhengquan Wu, Christoph A. Reichel, Christoph Walz, Martin Canis, Kristian Unger, Philipp Baumeister, Min Pan, Olivier Gires

**Affiliations:** 1 Department of Otorhinolaryngology, Head and Neck Surgery, Grosshadern Medical Center, Ludwig-Maximilians-University, Munich, Marchioninistr. 15, 81377 Munich, Germany; 2 State Key Laboratory of Oncogenes and Related Genes, Department of Oncology, Shanghai General Hospital, Shanghai Jiao Tong University School of Medicine, 100 Haining Road, Shanghai, China; 3 Research Unit Radiation Cytogenetics, Helmholtz Zentrum München, German Research Center for Environmental Health GmbH, Neuherberg, Germany; 4 Clinical Cooperation Group “Personalized Radiotherapy in Head and Neck Cancer”, Helmholtz Zentrum München, German Research Center for Environmental Health GmbH, Neuherberg, Germany; 5 Department of Radiation Oncology, University Hospital, LMU Munich, Munich, Germany; 6 Institute of Anatomy and Experimental Morphology, University Cancer Center Hamburg, University Medical Center Hamburg-Eppendorf, 20246 Hamburg, Germany; 7 Research Department Cell and Gene Therapy, Department of Stem Cell Transplantation, University Medical Center Hamburg-Eppendorf, 20246 Hamburg, Germany; 8 Institute of Pathology, Faculty of Medicine, LMU Munich, Munich, Germany; 9 Department of Otorhinolaryngology, The First Affiliated Hospital of Chongqing Medical University, Yuzhong District, Chongqing, China

**Keywords:** EGFR, ITGB4, EMT, HNSCC, EGFR-mediated invasion

## Abstract

**Background:**

Epidermal growth factor receptor (EGFR) is both a driver oncogene and a therapeutic target in advanced head and neck squamous cell carcinoma (HNSCC). However, response to EGFR treatment is inconsistent and lacks markers for treatment prediction. This study investigated EGFR-induced epithelial-to-mesenchymal transition (EMT) as a central parameter in tumor progression and identified novel prognostic and therapeutic targets, and a candidate predictive marker for EGFR therapy response.

**Methods:**

Transcriptomic profiles were analyzed by RNA sequencing (RNA-seq) following EGFR-mediated EMT in responsive human HNSCC cell lines. Exclusive genes were extracted via differentially expressed genes (DEGs) and a risk score was determined through forward feature selection and Cox regression models in HNSCC cohorts. Functional characterization of selected prognostic genes was conducted in 2D and 3D cellular models, and findings were validated by immunohistochemistry in primary HNSCC.

**Results:**

An EGFR-mediated EMT gene signature composed of *n* = 171 genes was identified in responsive cell lines and transferred to the TCGA-HNSCC cohort. A 5-gene risk score comprising DDIT4, FADD, ITGB4, NCEH1, and TIMP1 prognosticated overall survival (OS) in TCGA and was confirmed in independent HNSCC cohorts. The EGFR-mediated EMT signature was distinct from EMT hallmark and partial EMT (pEMT) meta-programs with a differing enrichment pattern in single malignant cells. Molecular characterization showed that ITGB4 was upregulated in primary tumors and metastases compared to normal mucosa and correlated with EGFR/MAPK activity in tumor bulk and single malignant cells. Preferential localization of ITGB4 together with its ligand laminin 5 at tumor-stroma interfaces correlated with increased tumor budding in primary HNSCC tissue sections. In vitro, ITGB4 knock-down reduced EGFR-mediated migration and invasion and ITGB4-antagonizing antibody ASC8 impaired 2D and 3D invasion. Furthermore, a logistic regression model defined ITGB4 as a predictive marker of progression-free survival in response to Cetuximab in recurrent metastatic HNSCC patients.

**Conclusions:**

EGFR-mediated EMT conveyed through MAPK activation contributes to HNSCC progression upon induction of migration and invasion. A 5-gene risk score based on a novel EGFR-mediated EMT signature prognosticated survival of HNSCC patients and determined ITGB4 as potential therapeutic and predictive target in patients with strong EGFR-mediated EMT.

**Supplementary Information:**

The online version contains supplementary material available at 10.1186/s12943-022-01646-1.

## Background

Locally advanced head and neck squamous cell carcinomas (HNSCC) account for 50% of all HNSCC and are characterized by poor prognosis with 5-years overall survival (OS) below 35% [1–3]. The prognosis of HNSCC patients is negatively impacted by field cancerization of large areas of the epithelium that is induced by long-term tobacco abuse, habitually in combination with increased alcohol consumption. Alternatively, HNSCC are induced by chronic infection with high-risk strains of human papillomavirus (HPV) [4]. HNSCC patients frequently suffer from local and regional recurrences, lymph node metastases, and high resistance to singular or combinatorial treatments with radiation and chemotherapy. Signaling pathways involved in the regulation of HPV-negative HNSCC include gain-of-function mutations in epidermal growth factor receptor (EGFR), NOTCH, phosphatidyl-inositol-3-phosphate kinase (PI3KCA) [5, 6]. Dysregulated EGFR expression and an ensuing impact on proliferation, EMT, migration, invasion, and angiogenesis have led to the implementation of EGFR-targeting drugs in the treatment of HNSCC patients [7]. These drugs include anti-EGFR therapeutic antibody Cetuximab, tyrosine kinase inhibitors, and phosphatidylinositol-3-kinase (PI3K) inhibitors. Cetuximab has approval for the treatment of advanced HNSCC [7], but therapy remains confined to palliative treatment of advanced disease stages rather than being intended in curative settings. Furthermore, therapy response to Cetuximab is limited, patients develop resistances, and predictive biomarkers supporting an informed decision-making in treatment planning are not available. Therefore, a deeper understanding of EGFR-mediated progression of HNSCC and of factors potentially defining treatment response are in great demand.

Genetic and transcriptomic profiling revealed an outstanding heterogeneity of primary HNSCC [4–6, 8] that inversely correlated with survival [9]. Basal, mesenchymal-enriched, classical epithelial-like, and atypical molecular subtypes were differentiated by bulk transcriptomic analyses in TCGA [5]. Tumor cell signatures were defined by single cell RNA-sequencing (scRNA-seq) and were related to epithelial differentiation, cell cycle, cell stress, hypoxia, and a partial epithelial-mesenchymal transition (pEMT) [10]. EMT is a trans-differentiation program co-opted by tumor cells that fosters dissemination, stemness features, immune evasion, and treatment resistance [11–16]. scRNA-seq resulted in a refinement of the four molecular subtypes identified in TCGA to a malignant-basal subtype incorporating tumors with a partial EMT signature (pEMT), a classical, and an atypical subtype. The former mesenchymal subtype was shown to arise from an increased presence of cancer-associated fibroblasts (CAFs) and was thus removed [10]. Decoupling EMT signatures of cancer cells and CAFs, Tyler and Tirosh identified three EMT signatures across hundreds of cancers and showed that while EMT signatures were not associated with metastases in most tumors, such association was observed in HNSCC [10, 17]. pEMT was strongly correlated with the malignant-basal HNSCC subtype [10], which is itself associated with enhanced EGFR expression and activation [18, 19]. Transfer of the scRNA-seq-based pEMT 15-gene common signature to bulk sequencing data from large HNSCC cohorts demonstrated its prognostic value and an association with the canonical EMT transcription factor SLUG [14].

EMT and pEMT regulation in HNSCC is governed by central signaling pathways including TGFβR, EGFR, NOTCH, and WNT [20]. Interactions of CAFs with tumor cells in the periphery of tumor areas were described to induce pEMT through TGFβ/TGFβ-R signaling [10]. Our group demonstrated a dual role of EGFR signaling in the regulation of proliferation and EMT in HNSCC, the latter one being achieved through ERK1/2 activation. EGFR-mediated EMT was dependent on treatment with high-dose EGF that upheld strong ERK1/2 activity, whereas low-dose treatment induced mild proliferation [21]. Additionally, the soluble extracellular domain of the marker of epithelial differentiation EpCAM termed EpEX, which is generated through regulated intramembrane proteolysis [22], was identified as a novel ligand of EGFR that induces a mild proliferation and counteracts EGFR-mediated EMT in HNSCC [21]. Eventually, EpEX was recognized as a novel ligand of EGFR in HNSCC, colon/colorectal carcinoma, and mesenchymal stem cells [21, 23–25]. Effects of the EpEX-EGFR axis on gene transcription and cellular functions are only beginning to be understood. In colorectal cancer, EpEX binding to EGFR stabilizes programmed-death ligand 1 (PD-L1) expression via inhibition of the transcription factor forkhead O3a (FOXO3a) [23]. Antagonizing EpEX in combination with an anti-PD-L1 antibody efficiently inhibited tumor formation in xenografted mice and enhanced the recruitment and activation of CD8^+^ T cells [23]. Molecular mechanisms of EpEX-mediated inhibition of EMT in HNSCC cells remain unexplored.

These molecular processes bear clinical relevance, as EGFR^low^/EpCAM^high^ HNSCC were characterized by an outstandingly good overall and disease-free survival (OS and DFS), whereas EGFR^high^/EpCAM^low^ tumors displayed very poor OS and DFS [21]. Hence, EGFR-mediated EMT may trigger molecular and functional changes relevant to HNSCC progression, shape the response to Cetuximab treatment, and help defining patients who would benefit from Cetuximab.

In the present study, a transcriptomic map of EGF-regulated genes was generated in responsive cell lines, determining an EGFR-mediated EMT gene signature. Gene signature transfer to large HNSCC cohorts allowed to identify a 5-gene prognostic signature and integrin β4 as a promising target to suppress EGF-induced cell invasion and predict tumor budding and response to Cetuximab.

## Results

### Transcriptome analysis of EpEX- and EGF-induced EGFR activation in HNSCC cell lines

**Fig. 1:**
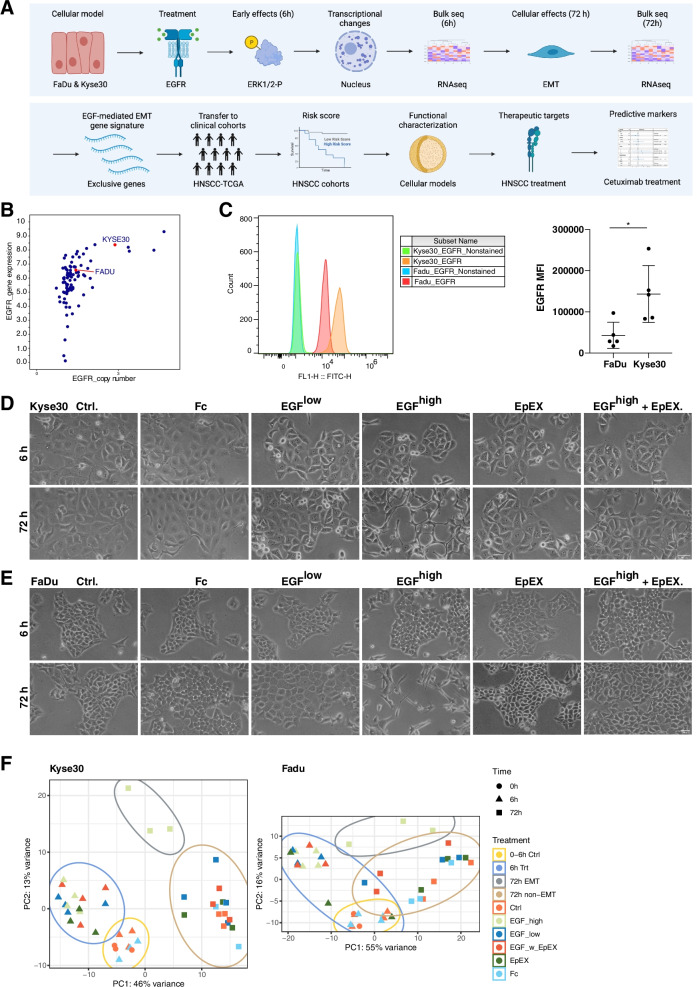
RNA-seq analysis of EGFR-mediated EMT. **A** Workflow: Kyse30 and FaDu cells were treated with EGF and EpEX inducing proliferation or EMT, respectively. Early transcriptional changes were assessed after 6 h and transcriptional differences associated with cellular effects after 72 h. EGFR-mediated EMT-associated genes serve to define prognostic gene signatures in clinical cohorts. Potential therapeutic and predictive markers in the EGFR-mediated EMT gene signature are explored via functional characterization and bioinformatic approaches. **B** Copy number variation and gene expression values of cell lines of the upper aerodigestive tract (esophageal carcinoma and HNSCC) were extracted from CCLE and are depicted as dot plot. **C** Kyse30 and FaDu cells were stained with EGFR-specific antibodies in combination with FITC-labeled secondary antibody. Shown are representative histograms (left panels) and mean expression values with SD of *n* = 5 independent experiments; * *p*-value < 0.05 (t-test). **D-E** Representative cytological pictures of Kyse30 and FaDu cells treated with the indicated components at 6 h and 72 h are shown (*n* = 4 independent experiments). Ctrl.: control treatment under serum-free conditions; Fc: recombinant immunoglobulin Fc region; EGF^low^: 1.8 nM EGF; EGF^high^: 9 nM EGF; EGF^high^ + EpEX: 9 nM EGF in combination with 50 nM EpEX-Fc. Scalebars represent 100 µm. **F** Principal component analysis of 3´-RNA-seq samples in Kyse30 and FaDu cells with the indicated treatments are shown (*n* = 4)

Kyse30 squamous cell esophageal carcinoma cells and FaDu squamous cell pharyngeal carcinoma were chosen to delineate effects of EpEX and EGF on EGFR signaling based on their responsiveness to EGFR-mediated EMT. Early effects of treatment have been observed at the level of ERK1/2 phosphorylation up to six hours, whereas morphologic and functional changes occurred after 48-72 h [21]. Bulk 3´RNA-seq transcriptome analysis of 6 and 72 h treatments established an EGFR-mediated EMT gene signature, which formed the basis of a signature transfer to clinical cohorts to assess prognostic, therapeutic, and predictive markers (Fig. 1A).

FaDu cells have neither amplifications nor mutations in *EGFR* [26], while Kyse30 show amplification of *EGFR* without mutations [27]. Copy number variations of Kyse30 (CNV + 1) and FaDu cells (CNV + 0) were assessed in datasets of the cancer cell line encyclopedia (CCLE) (Supplementary Fig. 1). CNV correlated with increased *EGFR* gene expression in Kyse30 compared to FaDu (Fig. 1B) and a 3.2-fold higher expression of EGFR in Kyse30 cells compared to FaDu cells was observed (Fig. 1C).

Serum-starved Kyse30 and FaDu cells were treated with EpEX-Fc (50 nM), EGF^low^ (1.8 nM), EGF^high^ (9 nM), or a combination of EpEX-Fc and EGF^high^. As controls, cells remained either untreated under serum-free conditions (controls for EGF treatment) or were treated with recombinant immunoglobulin Fc-region that served as control for EpEX-Fc. Fc allowed to express EpEX-Fc primarily in a dimeric form, which represents its natural state [21, 28]. Treatment with EGF^high^ resulted in the induction of a mesenchymal morphology after 72 h and co-treatment with EpEX inhibited EGF^high^-induced EMT (Fig. 1D-E). Principal component analysis showed distinct clustering of 0 and 6 h control-treated cells (0–6 h Ctrls.), 6 h samples independently of the treatment (6 h Treatment), 72 h treatments (72 h non-EMT), except EGF^high^-treated cells, which clustered separately (72 h EMT) (Fig. 1F).

### EGF^high^ induces sustained transcriptional activation

Gene set enrichment analysis (GSEA) was conducted for each treatment condition compared to controls, *i.e.* serum withdrawal in absence or presence of Fc. Significantly enriched gene ontology (GO)-terms were conserved for all four treatment modalities at 6 h and they primarily addressed ribosome biogenesis in Kyse30 cells and cell migration and motility in FaDu cells (Supplementary Fig. 2A). At 72 h, significantly enriched GO-terms were only identified for EGF^high^ treatment in Kyse30 cells, and for EGF^high^ and EGF^low^ treatments in FaDu cells. Significantly suppressed GO-terms in Kyse30 cells were related to DNA replication and nuclear division, whereas activated terms were related to keratinization, vesicle-mediated transport and cytokine signaling. Activated GO-terms in FaDu cells covered cell adhesion, cytoskeleton organization, and wound healing, while cell cycle-associated GO-terms were suppressed (Supplementary Fig. 2A). A similar activation of ribosome biogenesis at 6 h and a suppression at 72 h was observed in Kyoto encyclopedia of genes and genomes (KEGG) terms for Kyse30 cells. KEGG term “DNA replication” was suppressed in FaDu cells at 72 h, while KEGG terms significantly regulated at 6 h were heterogeneous and primarily associated with cytokine signaling (Supplementary Fig. 2B).

**Fig. 2:**
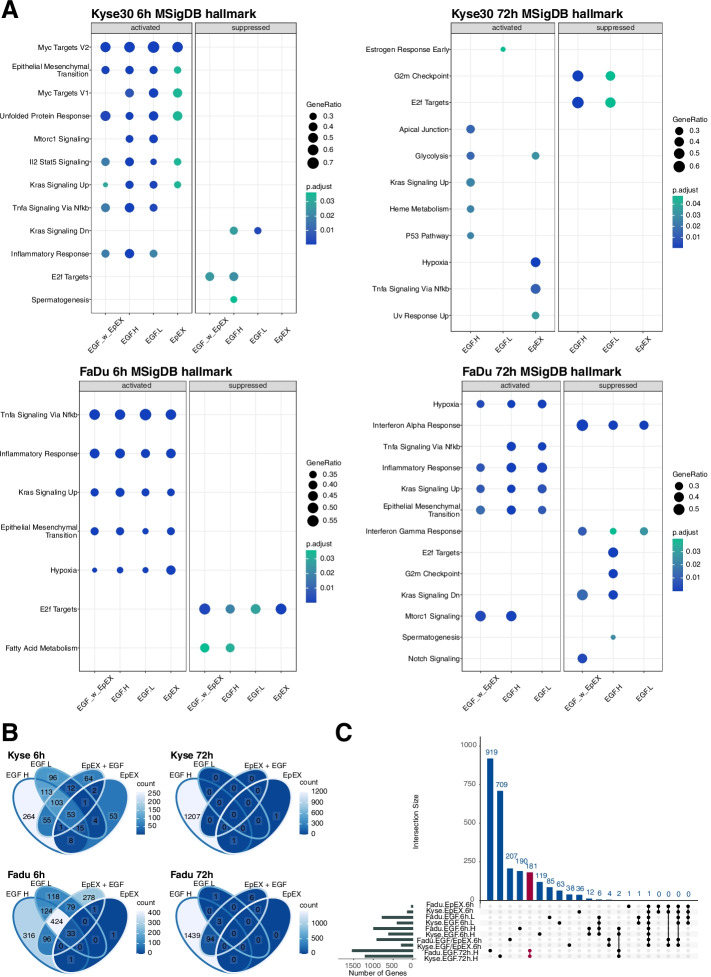
EGF- and EpEX-treatment associated GSEA and DEGs. **A** GSEA were performed for Kyse30 and FaDu cells at 6 h and 72 h after the indicated treatment (EGF^low^: EGFL, EGF^high^: EGFH, EpEX, EGF^high^ with EpEX: EGF_w_EpEX) using MSigDB hallmark gene sets. Significantly activated or suppressed hallmarks are depicted with gene ratios and adjusted p-values. **B** Venn diagram of DEGs (|log2FC|> 0.5; adjusted *p*-value ≤ 0.05) at 6 h and 72 h for Kyse30 and FaDu cells under indicated treatments. **C** Upset plot displays exclusive DEGs in combinations (connected bullet points). Numbers of exclusive DEGs are indicated

Molecular signatures database (MSigDB) hallmark gene sets of biological processes or states from the BROAD institute were further chosen for GSEA. MSigDB hallmarks are composed of genes identified in well-characterized, ground-truth-based cellular and animal systems for 50 major biological processes in which the selected genes display an interrelated expression. “Epithelial Mesenchymal Transition” and “Kras Signaling Up” were activated in both cell lines at 6 h for all treatments (Fig. 2A). At 72 h, Kyse30 cells were characterized by activated “Glycolysis” (EGF^high^ and EpEX), “Kras Signaling Up”, “Heme metabolism”, and “P53 Pathway” (EGF^high^), and by suppressed “G2M Checkpoint” and “E2F Targets” (EGF^high^ and EGF^low^). Suppression of “E2F Targets” and “G2M Checkpoint” was confirmed in FaDu cells treated with high-dose EGF. Additionally, FaDu cells activated MSigDB hallmarks “Inflammatory Response”, “Kras Signaling Up”, and “Epithelial Mesenchymal Transition” following treatment with EGF^high^, EGF^low^, and a combination of EGF^high^ with EpEX (Fig. 2A).

Regulated MSigDB hallmarks were comparable across all four cell treatments at 6 h and identified an induction of EMT by all treatments. However, independent, published results from our group identified morphologic, molecular, and functional traits of EMT only following EGF^high^ treatment using morphology features, EMT-TF gene expression patterns, and migration behavior [21]. Therefore, differentially expressed genes (DEGs) of treated cells versus controls were identified to compare the strength of gene regulation across treatments (|log2FC|> 0.5; adjusted *p*-value ≤ 0.05). EGF^high^ treatment resulted in highest numbers of DEGs and EpEX treatment in lowest numbers of DEGs after 6 h (Fig. 2B, left panels). These differences in gene regulation were exacerbated at 72 h, when EGF^high^ induced *n* = 1208 and *n* = 1536 DEGs in Kyse30 and FaDu cells, respectively (Fig. 2B). All other treatments resulted in no DEGs (Kyse30, EGF^low^ and EGF^high^ + EpEX; FaDu, EpEX), few DEGs (Kyse30, EpEX *n* = 2; FaDu, EGF^low^ *n* = 4), or moderate regulation (FaDu, EGF^high^ + EpEX *n* = 103) (Fig. 2B).

To filter genes that are potentially associated with the differential outcome of EpEX, EGF^low^, and EGF^high^ treatments, DEGs exclusively regulated by each treatment in Kyse30 and FaDu cells were visualized in an upset plot (Fig. 2C). Numbers of exclusive DEGs for each separate treatment at 6 h ranged from *n* = 1 (FaDu cells treated with EpEX) to a maximum of *n* = 207 (FaDu cells treated with EGF^high^ + EpEX). Highest numbers of exclusive DEGs were observed following treatment with EGF^high^ after 72 h in FaDu (*n* = 919) and Kyse30 cells (*n* = 709), respectively. Thus, induction of EMT by EGF^high^ is associated with sustained and strong gene regulation compared with treatments that failed to induce morphologic and functional EMT.

### EpEX induced gene transcription

**Fig. 3:**
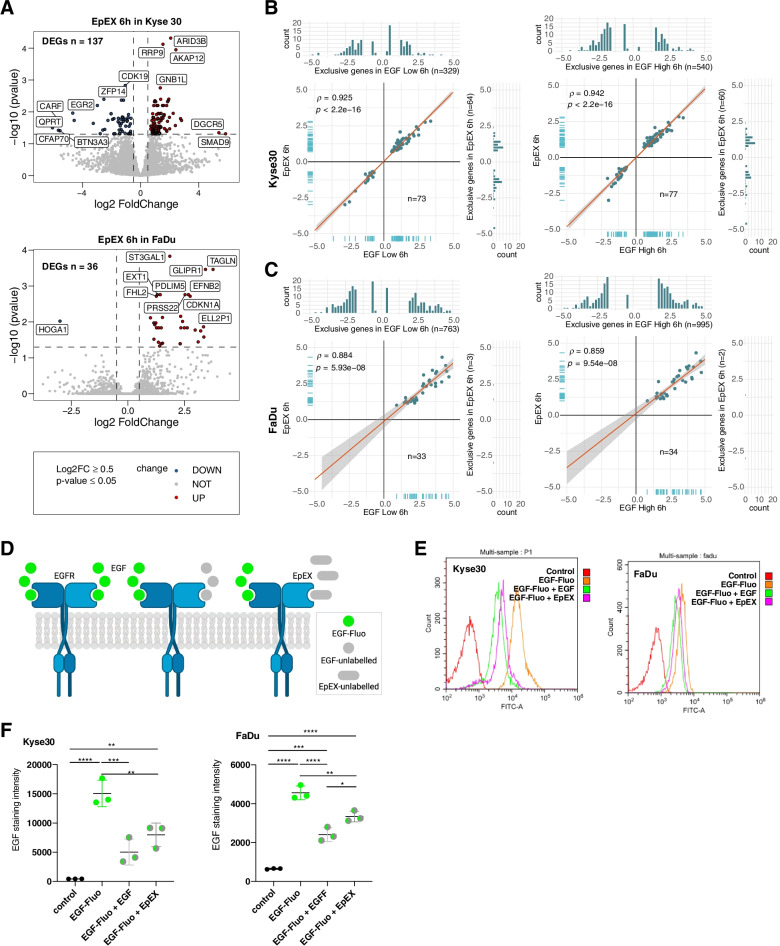
EpEX regulates gene expression and competes with EGF for binding to EGFR. **A** Volcano plots of DEGs in Kyse30 and FaDu cells 6 h after EpEX treatment. Numbers of DEGs for each cell line are indicated (|log2FC|> 0.5; adjusted *p*-value ≤ 0.05). **B-C** Shared and exclusive DEGs between EpEX, EGF^low^, and EGF^high^ are depicted in scatter/bar plots with Spearman correlation and p-values. **D** Schematic representation of the EGF/EpEX competition assay. Cells were incubated with fluorescence-labeled EGF alone or in combination with non-labeled EGF or EpEX in equimolar conditions. **E–F** Fluorescence of Kyse30 and FaDu cells incubated with fluorescence-labeled EGF and combinations with non-labeled EGF and EpEX was quantified by flow cytometry. **E** Representative histograms of EGFR staining in a competition assay in Kyse30 and FaDu cells. **F** Mean values with SD of *n* = 3 independent experiments are shown. *P*-values from one-way ANOVA with Tukey´s multiple comparison test. * ≤ 0.05; ** ≤ 0.01; *** ≤ 0.001; **** ≤ 0.0001. Control = unstained cells; EGF-Fluo: fluorescence-labeled EGF; EGF-Fluo + EGF: fluorescence-labeled EGF plus unlabeled EGF; EGF-Fluo + EpEX: fluorescence-labeled EGF plus EpEX

Effects of EpEX on gene transcription were compared to EGF-dependent regulation at 6 h, since no or only two DEGs were retrieved after 72 h in Kyse30 and FaDu cells, respectively (Fig. 2B). EpEX-Fc induced *n* = 137 and *n* = 36 DEGs in Kyse30 and FaDu cells, respectively (Fig. 3A). In Kyse30 cells, *n* = 73/137 and *n* = 77/137 EpEX DEGs were shared with EGF^low^ and EGF^high^, respectively. Shared DEGs were similarly regulated with Spearman ρ-values of 0.925 and 0.942 and high statistical significance (Fig. 3B). In FaDu cells, *n* = 33/36 and *n* = 34/36 EpEX DEGs were shared with EGF^low^ and EGF^high^, respectively, and ρ-values were 0.884 and 0.854 (Fig. 3C). Hence, none of the shared DEGs were counter-regulated or differed substantially regarding the magnitude of regulation. In Kyse30 cells, EpEX induced *n* = 64 and *n* = 60 exclusive DEGs compared to EGF^low^ and EGF^high^, respectively. Exclusive EpEX DEGs were very restricted in FaDu cells (*n* = 3 and *n* = 2). Furthermore, EGF^low^ and EGF^high^ treatments induced a substantially higher number of exclusive DEGs compared to EpEX-Fc in both cell lines (Kyse30: EGF^low^ *n* = 329, EGF^high^ *n* = 540; FaDu: EGF^low^ *n* = 763, EGF^high^ *n* = 995) (Fig. 3B-C).

We concluded that EpEX-Fc is a weak ligand of EGFR that regulates a subset of EGF-dependent genes and few unique genes. Thus, EpEX-Fc-dependent repression of EGFR-mediated EMT is most likely not the result of a transcriptional repression but of a competition with EGF for binding to EGFR. To test this hypothesis, Kyse30 and FaDu cells were incubated with fluorescently labeled EGF in absence or presence of equimolar amounts of unlabeled EGF or EpEX-Fc, and fluorescence intensities were measured by flow cytometry (Fig. 3D). Incubation with labeled EGF increased mean fluorescence intensities (MFI) in both cell lines (38.8-fold and 7.0-fold in Kyse30 and FaDu, respectively). Differences in fluorescence induction were congruent with EGFR expression levels on the respective cell line. Co-treatment with unlabeled EGF reduced the fluorescence by 66.7% and 47.2% in Kyse30 and FaDu cells, respectively. Co-treatment with EpEX-Fc reduced the fluorescence by 47.1% and 28.8% in Kyse30 and FaDu cells, thus confirming a competitive binding of EGF and EpEX to EGFR (Fig. 3E-F). Hence, EpEX is a ligand of EGFR that induces lesser transcriptional changes than EGF and competes with EGF for binding to EGFR.

### EGFR-mediated EMT differs from EMT hallmark and pEMT signatures

**Fig. 4:**
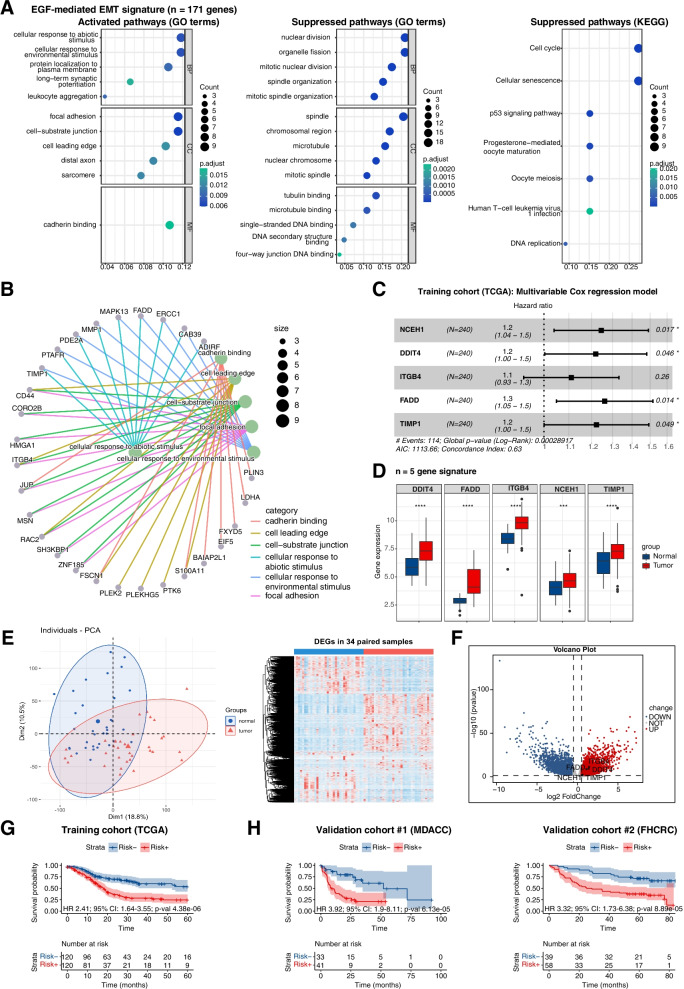
EGFR-mediated EMT-dependent 5-gene prognostic signature for HNSCC. **A** Genes of the EGFR-mediated EMT signature (*n* = 171) were subjected to an over-representation analysis. Significantly activated and suppressed pathways in GO and KEGG are depicted with gene counts and adjusted p-value. **B** Genes from the enriched GO-terms are depicted in a gene-concept network. **C** Forest plot of the multivariable Cox PH regression model incorporating the 5-gene signature in the training cohort (TCGA) of *n* = 240 HPV-negative HNSCC with event numbers, log-rank p-value, AIC, and concordance index. **D** Gene expression of DDIT4, FADD, ITGB4, NCEH1, and TIMP1 in normal mucosa (*n* = 34) and HNSCC (*n* = 238) of the training cohort (TCGA); *p*-values *** ≤ 0.001; **** ≤ 0.0001. **E** Principal component analysis and hierarchical clustering of the top 25% most strongly regulated DEGs for matched pairs of normal mucosa and HNSCC of the TCGA cohort are shown (*n* = 34). **F** Volcano plot of DEGs from matched pairs of normal mucosa and HNSCC of the TCGA cohort (*n* = 34; |log2FC|> 0.5, *p*-value ≤ 0.05). Genes of the 5-gene signature are significantly up-regulated DEGs. **G** Stratification of HPV-negative HNSCC of the training cohort (TCGA; *n* = 240) with a 5-gene signature-based risk score (median cut-off) for overall survival (time in months). Numbers at risk, HR, 95% CI, and p-value are indicated in the Kaplan–Meier survival curve. **H** Validation of the risk score in the MD Anderson Cancer Center (MDACC; *n* = 74) and the Fred Hutchinson Cancer Research Center (FHCRC; *n* = 97) HNSCC cohorts. Median cut-off threshold of the training cohort served to dichotomize the MDACC and FHCRC cohorts (risk − , risk +) for overall survival. Numbers at risk, HR, 95% CI, and p-value are indicated in the Kaplan–Meier survival curve

Intersected exclusive DEGs induced by EGF^high^ in Kyse30 and FaDu cells were extracted (*n* = 181, Fig. 2C) and 171 genes were similarly regulated in both cell lines. This EGFR-mediated EMT signature (*n* = 171) was subjected to an over-representation analysis (ORA). GO-terms activated in the EGFR-mediated EMT signature were related to cell migration and invasion (“focal adhesion”, “cell-substrate junction”, “cell leading edge”, and “cadherin binding”) (Fig. 4A, left panel). Suppressed GO-terms were related to DNA replication and cell division, pinpointing a reduced cell proliferation following EGFR-mediated EMT (Fig. 4A, middle panel). This finding was corroborated by the suppressed KEGG terms “cell cycle” and “DNA replication”. Additionally, EGFR-mediated EMT was associated with the KEGG terms of “cellular senescence” and “p53 signaling pathway” (Fig. 4A, right panel). Genes included in enriched GO-terms were extracted and linkages with GO-terms are depicted in a gene-concept network. HNSCC cancer stem cell (CSC) marker CD44, integrin beta 4 (ITGB4), and small GTPase Rac2 were linked to the terms “Cell leading edge”, “Cell-substrate junction”, and “Focal adhesion”, while further genes showed linkages to one or two GO-terms (Fig. 4B). Gene-concept networks of suppressed GO-terms and KEGG-terms are depicted in chord plots in Supplementary Fig. 3. Regulation of Polo-like kinase 1 (PLK1) and Kinesin family members was linked to numerous GO-terms related to cell cycle and nuclear division.

Analysis of overlapping and unique DEGs of the EGFR-mediated EMT signature with the MSigDB EMT hallmark and the pEMT [10] gene sets revealed only few overlapping genes (*n* = 7/171 (4%) and *n* = 4/171 (2.3%), respectively), whereas pEMT and EMT hallmark showed more similarity (*n* = 36/100 (36%)) (Supplementary Fig. 4A and Supplementary Table 1). These results suggested molecular heterogeneity across all three signatures potentially defining differing states of EMT. Two publicly available datasets (Gene Expression Omnibus accession numbers GSE23952 and GSE32254), which were used by Broad MSigDB for refining and validating their ‘EMT hallmark’ gene set, were used here as ground-truth to define EMT. Gene set variation analysis (GSVA) was conducted with the MSigDB hallmark gene set (*n* = 200 genes), the pEMT signature (*n* = 100 genes) [10], and the EGFR-mediated EMT signature (*n* = 171 genes). All three EMT signatures were significantly enriched in cells that had undergone EMT following TGFβ (GSE23952) and TNFα/TGFβ treatment (GSE32254) (Supplementary Fig. 4B).

Malignant cells (*n* = 2,176) from the scRNA-seq HNSCC dataset GSE103322 were subjected to a GSVA of the pEMT signature, the MSigDB hallmark gene set, and the EGFR-mediated EMT signature. Enrichment of the pEMT signature across all ten HNSCC patients was regarded as ground-truth in this analysis and GSVA results were highly comparable to original findings, which were re-computed in analogy to Puram et al*.* using *Seurat* R *AddModuleScore* (Supplementary Fig. 4C-D, Spearman *ρ* = 0.95, *p*-value 2.2e-16). Comparison of pEMT, EMT, and EGFR-mediated EMT signature enrichments at the single cell level showed overlap as well as differences across all three signatures. Cells enriched for the EGFR-EMT were more frequent across patients and were also detected in HNSCC that have not been deemed in pEMT (P6, P20). Furthermore, single cells within patients differed in the enrichment of EGFR-mediated EMT, whereas pEMT and EMT enrichment were more concurrent (see for example P5, P16, P22, and P28) (Supplementary Fig. 4C). Correlation analysis of all three EMT scores confirmed a higher concordance between pEMT and the MSigDB EMT hallmark and lower concordance with EGFR-mediated EMT (Supplementary Fig. 4D). Lastly, comparing pEMT, EMT, and EGFR-mediated EMT scores at the individual patient level confirmed a more homogeneous distribution of EGFR-mediated EMT across patients. However, a more heterogeneous degree of EGFR-mediated EMT was observed across single cells of a given patient, with two or three separate major sub-populations of single cells defined in violin plots (Supplementary Fig. 4E). Hence, EGFR-mediated EMT defines a meta-program in single malignant cells of HNSCC that is partly redundant yet distinct to pEMT and EMT.

### EGFR-mediated EMT-dependent prognostic risk score in HNSCC

The EGFR-mediated EMT signature was transferred to HPV-negative patients of the TCGA-HNSCC cohort [5] (*n* = 240; Supplementary Table 2) as training cohort to develop a prognostic risk score. Expression values for *n* = 170 genes from the EGFR-mediated EMT signature were available and subjected to univariate Cox regression analysis of the correlation to OS. Assuming that EGFR-mediated EMT is detrimental to the patients´ survival, up-regulated genes with a HR > 1 and down-regulated genes with HR < 1 were filtered (*n* = 53 genes). Following forward feature selection using *rbSurv*, a multivariate Cox regression model defined a 5-gene signature composed of NCEH1 (Neutral Cholesterol Ester Hydrolase 1), DDIT4 (DNA Damage Inducible Transcript 4), ITGB4, FADD (Fas-Associated protein with Death Domain), and TIMP1 (Tissue Inhibitor of Metaloproteinases 1) as prognostic risk score (Fig. 4C).

Gene expression of the 5-gene signature was assessed with HTSeq-counts for *n* = 238 HNSCC and *n* = 34 normal samples from TCGA. The expression of all five genes was induced following EGF^high^-treatment in Kyse30 and FaDu cells and were significantly up-regulated in HNSCC versus normal samples (Fig. 4D). Thirty-four matched pairs of normal tissue and HNSCC were identified within TCGA, which separated in two major clusters in a PCA and a hierarchical clustering heatmap based on the top 25% most highly expressed genes (Fig. 4E). All five genes of our prognostic signature (NCEH1, DDIT4, ITGB4, FADD, and TIMP1) were significantly up-regulated DEGs from tumors in matched pairs of HNSCC and normal tissue from TCGA data (Fig. 4F; |log2FC|> 0.5, *p*-value ≤ 0.05).

HPV-negative TCGA-HNSCC patients were stratified according to the 5-gene signature-based risk score (median threshold) and survival probabilities depicted in Kaplan–Meier (KM) curves. High risk scores correlated with significantly reduced 5-year OS (Fig. 4G; HR 2.41, 95% CI: 1.64–3.55, *p*-value = 4.38e-06). mRNA coefficients and the median threshold of the prognostic model trained in the TCGA cohort were transferred to validation cohorts (MDACC-HNSCC, *n* = 74; FHCRC, *n* = 97; Supplementary Table 2) and confirmed the correlation of the EGFR-mediated EMT-based risk score with poor OS (Fig. 4H; MDACC: HR 3.92, 95% CI: 1.9–8.11, *p*-value = 6.13e-05; FHCRC: HR 3.32, 95% CI: 1.73–6.38, *p*-value = 8.89e-05). Thus, the EGFR-mediated EMT-based risk score prognosticates OS in HNSCC.

The prognostic value of the EGFR-mediated EMT signature to predict OS was compared to published EMT signatures for HNSCC. The MSigDB EMT hallmark signature, the HNSCC pEMT signature by Puram et al*.* and the HNSCC EMT signatures by Jung et al*.* and Vallina et al*.* served as comparisons [10, 29, 30]. Except for the Vallina et al*.* signature that is composed of six genes, which were extracted in a meta-analysis of eight independent cohorts, all remaining larger signatures were subjected to feature selection using *rbsurv* and multivariate Cox regression model within the TCGA HNSCC cohort. The MSigDB EMT hallmark signature (*n* = 200 genes) retrieved a 4-gene risk score (NT5E, PVR, COL8A2, and APLP1), the pEMT signature (*n* = 100 genes) a 5-gene risk score (IGFBP7, EMP3, SLC39A14, CALU, and SLC38A5), and the Jung et al*.* signature (*n* = 82 genes) a 2-gene risk score (GLT8D2 and COL6A1). Only the pEMT- and the EGFR-mediated EMT-based risk scores showed a significant Log-Rank p-value in the multivariate Cox model, with a concordance index of the pEMT-based risk score that was slightly inferior to the EGFR-mediated EMT-based risk score (0.59 vs. 0.63) (Supplementary Fig. 5A-D and Fig. 4C). Stratification of the patients according to the median risk score for each model showed a significant prognosis of OS for the EGFR-mediated EMT-, the MSigDB EMT hallmark- and the pEMT-based risk scores in KM curves (EMT hallmark: HR 1.46; 95% CI 1.01–2.11; *p* = 0.044; pEMT: HR 1.69; 95% CI 1.16–2.46; *p* = 0.0054) (Supplementary Fig. 5A-D and Fig. 4C). Analysis of the area under the curve (AUC) for false and true positives for risk scores generated from all five models confirmed superior performances of the EGFR-mediated EMT- and the pEMT-based risk scores to prognosticate 3- and 5-year OS (Supplementary Fig. 5E).

### ITGB4-associated gene expression in HNSCC

**Fig. 5:**
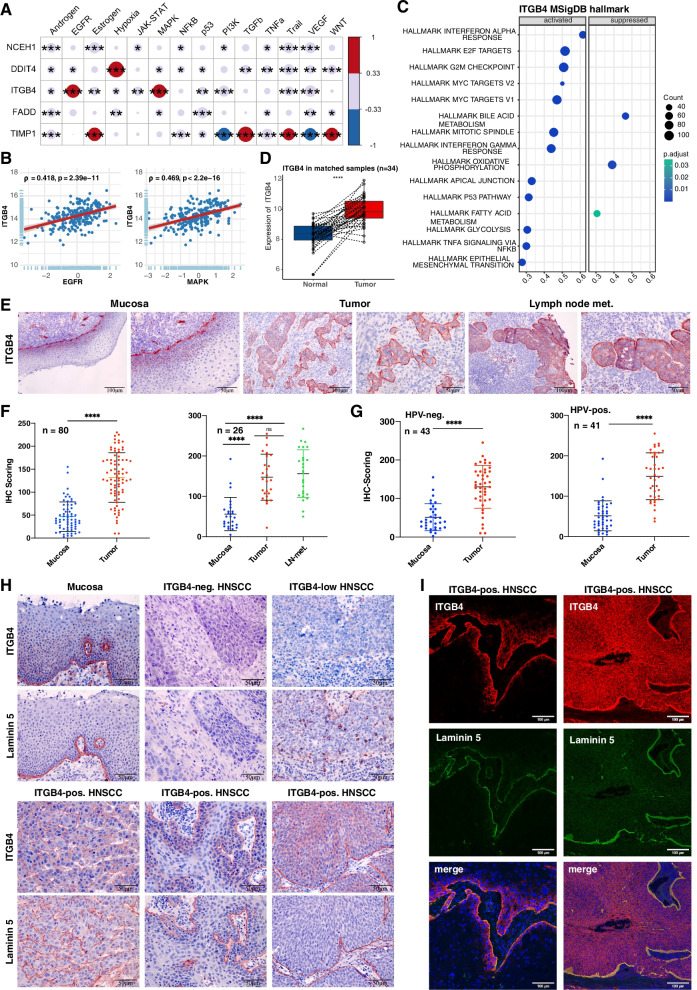
Integrin beta 4 (ITGB4) expression in HNSCC. **A** Pathway activities were inferred in HPV-negative TCGA-HNSCC samples using PROGENy (*n* = 240). Spearman correlations and p-values of the 5-gene signature with pathway activities are depicted. * ≤ 0.05; ** ≤ 0.01; *** ≤ 0.001. **B** Scatter plots of ITGB4 correlations with EGFR and MAPK pathways in HPV-negative HNSCC of TCGA patients (*n* = 240) are shown with Spearman correlation and p-value. **C** ITGB4-correlated genes were subjected to a GSEA using MSigDB hallmark gene sets. Top 15 regulated hallmarks are depicted with gene counts and adjusted *p*-values. **D** ITGB4 expression was compared in matched pairs of normal mucosa and HPV-negative HNSCCs of TCGA (*n* = 34). Matched expression values are shown in a box plot whiskers graph (t-test **** ≤ 0.0001). **E** Representative examples of ITGB4 expression in normal mucosa (*n* = 64), primary tumor (*n* = 80) belonging to *n* = 84 patients, and in triplets including lymph node metastases (*n* = 26) from HPV-neg. and -pos. HNSCC of the LMU cohort. **F-G** IHC scoring of ITGB4 protein expression is shown in scatter dot plots with means and SD for all samples and stratified according to HPV-status. Ns: not significant, **** ≤ 0.0001 (t-test and One-way ANOVA). **H** Representative examples of ITGB4 and laminin 5 expression in consecutive sections of normal mucosa and HNSCC are shown. **I** Double immunofluorescence staining of ITGB4 (red), laminin 5 (green), and DAPI (blue) in HNSCC with edge-localized (left) or homogeneous ITGB4 expression (right)

NCEH1, DDIT4, ITGB4, FADD, and TIMP1 expressions in the TCGA cohort were correlated with major signaling pathway activities inferred using PROGENy (Pathway RespOnsive GENes for activity inference). PROGENy compiles ground-truth validated core responsive genes from 14 major cellular pathways, allowing to compute pathway activity scores from bulk and scRNA-seq datasets [31]. ITGB4 was significantly correlated with EGFR and mitogen-activated protein kinase (MAPK) pathway activities (Fig. 5A-B), which are both required for EGFR-mediated EMT [21]. ITGB4 co-regulated genes were identified by batch correlation analysis in the HPV-negative TCGA cohort. The highest Spearman correlation was observed for Integrin alpha 3 (ITGA3), which is a binding partner of integrin beta 1 (ITGB1) that forms a heterodimeric laminin receptor. LAMA3, LAMB3, and LAMC2, together encoding the ITGB4 ligand laminin 5, were in the top ten co-regulated genes and are part of the common pEMT gene signature [10] that prognosticates OS in HNSCC [14]. Focal adhesion-associated adapter protein paxillin was likewise strongly correlated to ITGB4 expression. Top ten genes that showed a counter-regulated expression to ITGB4 were more heterogeneous in function, including transcriptional activators/repressors and replication factors (CREG1, REPIN1, AFF3, WDSUB1, EID3), membrane-associated enzymes and functional proteins (CA5B, GULP1), methyltransferase 9, complement inhibitor SUSD4, and one gene of unclear function (C12ORF57) (Supplementary Table 3).

ITGB4-associated hallmarks were inferred via GSEA of all genes from TCGA-HNSCC ranked by their Spearman correlation with ITGB4 and using MSigDB hallmarks gene sets. Activated hallmarks were related to cell proliferation (“Hallmark of E2F targets”, “Hallmark of G2M checkpoint”, Hallmark mitotic spindle”), glycolysis, apical junction, TNF alpha signaling via NF kappa B, and EMT. Suppressed hallmarks referred to bile acid metabolism, oxidative phosphorylation, and fatty acid metabolism (Fig. 5C).

### ITGB4 and its ligand laminin 5 are up-regulated in HNSCC

Matched pairs of normal mucosa and HNSCC from the TCGA-HNSCC cohort confirmed a significant over-expression of ITGB4 mRNA in tumor samples (Fig. 5D). ITGB4 protein expression was analyzed in an in-house cohort of HPV-negative and -positive HNSCC patients (Supplementary Table 2). Primary HNSCCs (*n* = 80) in comparison to normal mucosa (*n* = 64) proved a significant over-expression of ITGB4 in tumors (Fig. 5E and F left panel). Representative IHC staining in Fig. 5E showed a strong expression of ITGB4 at the basal cell membrane adjacent to the basal lamina and in suprabasal cell layers of normal mucosa and the over-expression in tumor areas. Matched triplets (*n* = 26) of normal mucosa, primary tumor, and lymph node metastases demonstrated a significant over-expression of ITGB4 in primary tumors and lymph node metastases (Fig. 5E and F right panel). Stratification of patients according to the HPV-status showed a significant up-regulation of ITGB4 in HPV-negative (*n* = 43) and -positive (*n* = 41) patients (Fig. 5G).

ITGB4 and laminin 5 were assessed in serial sections of HNSCC with weak and strong ITGB4 expression. In normal mucosa, ITGB4 and laminin 5 were co-localized at the basal lamina and ITGB4 was additionally expressed in suprabasal cell layers (Fig. 5H). In ITGB4-negative or ITGB4^low^ HNSCC, laminin 5 was either absent or only expressed in non-malignant cells representing endothelial cells and leukocytes. Differing localization of ITGB4 was observed within tumor areas including an exclusive expression at the interface between tumor and non-malignant stromal tissue, a marginal expression at the edges of tumor areas, and a more homogeneous expression throughout tumor cells. Laminin 5 co-localized with ITGB4 at the tumor-stroma interface (Fig. 5H). Co-localization patterns of ITGB4 and laminin 5 were confirmed in double immunofluorescence staining (Fig. 5I).

Normal mucosa (*n* = 34), and HNSCC (*n* = 238) and matched pairs of normal mucosa and HNSCC (*n* = 34) from the TCGA cohort were assessed for the expression of integrin alpha 6 (ITGA6) and laminin 5 genes LAMA3, LAMB3, and LAMC2. Both sub-cohorts demonstrated a significant up-regulation of ITGA6, LAMA3, LAMB3, and LAMC2 in tumors (Supplementary Fig. 6A). ITGB4 and ITGA6, LAMA3, LAMB3, and LAMC2 showed a positive correlation in HNSCC of the TCGA cohort and in single malignant HNSCC cells (Supplementary Fig. 6B-C).

### ITGB4 correlates with EGFR activity in malignant single HNSCC cells

**Fig. 6:**
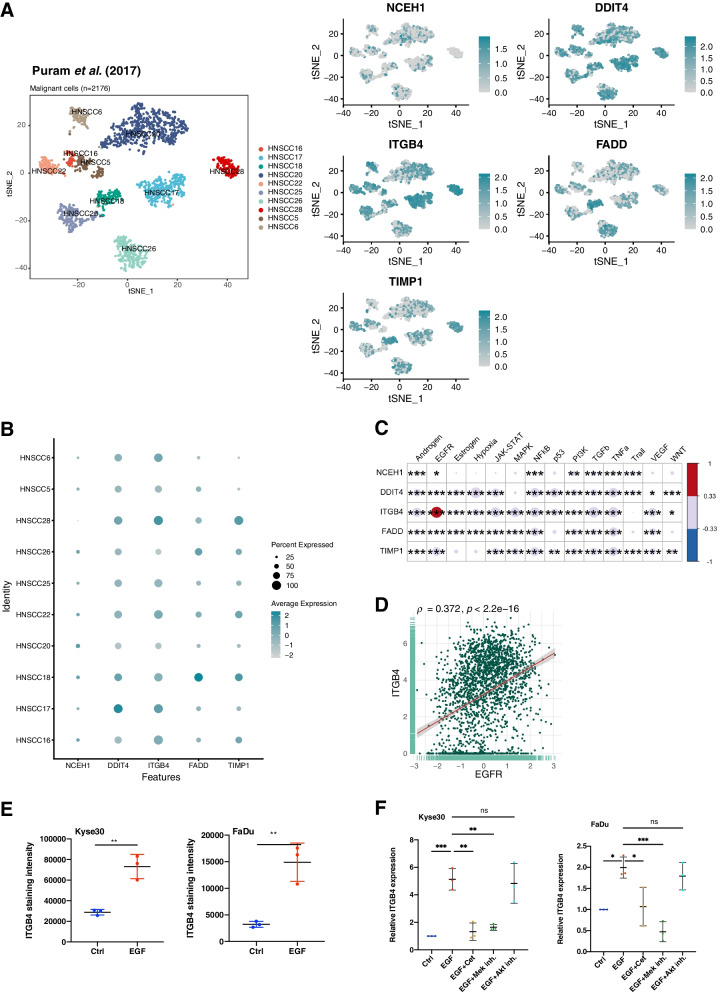
5-gene signature expression in malignant single HNSCC cells. **A** T-distributed stochastic neighbor embedding (t-SNE) plots of malignant single HNSCC cells (*n* = 2176; GSE103322; left plot). Expression of NCEH1, DDIT4, ITGB4, FADD, and TIMP1 are displayed in t-SNE plots in n = 2176 malignant single cells (right plots). **B** 5-gene signature expression is represented for individual patients with percent of malignant single cell expression and average expression values. **C** Pathway activities of malignant single cells from GSE103322 were inferred using PROGENy. Spearman correlations and p-values of the 5-gene signature with pathway activities are depicted. * ≤ 0.05; ** ≤ 0.01; *** ≤ 0.001. **D** Scatter plots of ITGB4 correlations with the EGFR pathway in *n* = 2176 malignant single cells from GSE103322 are shown with Spearman correlation and p-value. **E** ITGB4 cell surface protein expression is shown in Kyse30 and FaDu cells treated with 50 ng/mL EGF for 72 h. Mean values with SD are shown in scatter dot plots of *n* = 3 independent experiments. ** ≤ 0.01 (t-test). **F** ITGB4 mRNA expression in Kyse30 and FaDu cells treated with 50 ng/mL EGF or combinations of EGF with Cetuximab, MEK inhibitor, or AK inhibitor. Mean values with SD of qPCR measurements are shown in scatter dot plots after 72 h of the indicated treatment of *n* = 3 independent experiments. * ≤ 0.05; ** ≤ 0.01; *** ≤ 0.001 (one-way ANOVA)

The 5-gene signature expression was analyzed in malignant single HNSCC cell data from Puram et al*.* [10]. Ten oral cavity carcinomas with a total of *n* = 2176 single cell transcriptomes from the scRNA-seq dataset GSE103322 were included in the analysis (tSNE plot in Fig. 6A). Expression analysis of the 5-gene signature revealed highest expressions of ITGB4 and DDIT4 at the individual patient level and highest percentages of positive single cells (Fig. 6A and B).

NCEH1, DDIT4, ITGB4, FADD, and TIMP1 expression was correlated to pathway activities inferred in single cells using PROGENy. Highest significant correlation was observed for ITGB4 and the EGFR pathway activity (Fig. 6C-D). Additionally, ITGB4 expression was analyzed in scRNA-seq datasets of different cancer entities using TISCH (Tumor Immune Single-Cell Hub) [32]. TISCH is an online scRNA-seq database composed of *n* = 79 datasets and *n* = 2,045,746 cells, allowing for the expression analysis of genes of interest in malignant and non-malignant cells of *n* = 18 individual cancer types including HNSCC (http://tisch.comp-genomics.org/). Cancer entities with highest ITGB4 expression in single malignant cells were compared (log(TPM/10 + 1) > 0.5). ITGB4 was substantially expressed in single cells of basal cell carcinoma, cholangiocarcinoma (CHOL), colorectal carcinoma CCRC), glioma, HNSCC, non-small cell lung cancer (NSCLC), ovarian serous cystadenocarcinoma (OV), and pancreatic adenocarcinoma (PAAD). HNSCC were characterized by the highest ITGB4 expression in malignant single cells and the strongest differential expression compared to immune and stromal cells (Supplementary Fig. 7A, upper panel). Expression analyses in subtypes of immune and stromal cells showed additional expression of ITGB4 in endothelial cells, only (Supplementary Fig. 7B). ITGB4 expression was represented in violin plots for the three tumor entities with highest expression*, i.e.* HNSCC, PAAD, and CRC, confirming the strong and most selective expression in malignant HNSCC cells (Supplementary Fig. 8C). ITGA6, LAMA3, LAMB3, and LAMC2 followed the expression pattern of ITGB4 and was highest in malignant single HNSCC cells und most differential to immune, stromal, and other cells (Supplementary Figs. 8– 11).

Induction of ITGB4 cell surface expression by EGFR activation was validated using flow cytometry. ITGB4 protein expression at the plasma membrane was enhanced 2.53-fold and 4.62-fold in Kyse30 and FaDu cells upon treatment with EGF^high^, respectively (Fig. 6E). ITGB4 mRNA expression was induced by 5.12-fold and 1.99-fold in Kyse30 and FaDu cells upon EGF^high^ treatment. Cetuximab and MEK inhibitor co-treatment blocked this induction, whereas AKT inhibition had no impact on ITGB4 mRNA induction (Fig. 6F). Hence, ITGB4 is over-expressed and associated with enhanced EGFR and MAPK activity in malignant single cells of HNSCC.

### ITGB4 promotes migration and invasion

**Fig. 7:**
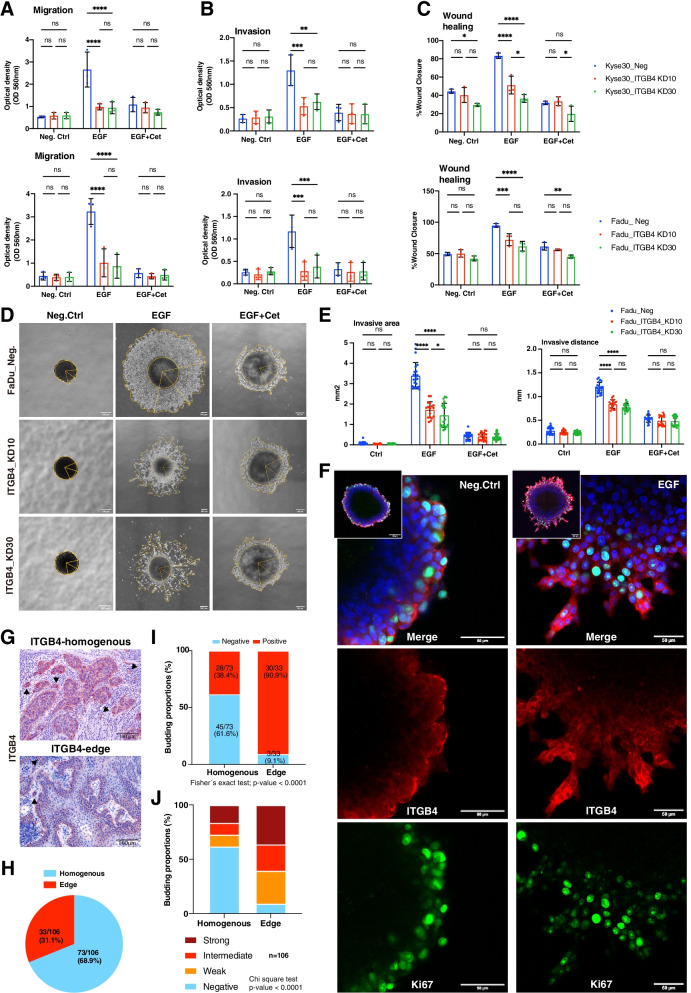
ITGB4 promotes migration and invasion of HNSCC cells. **A-B** Control and ITGB4 knock-down cells were analyzed in migration (**A**) and invasion assays with inlays coated with Matrigel (**B**) in a Boyden chamber. Mean and SD are shown in scatter dot plots of *n* = 3 independent experiments. * ≤ 0.05, ** ≤ 0.01; *** ≤ 0.001, **** ≤ 0.0001. **C** Control and ITGB4 knock-down cells were analyzed in wound healing assays. Wound closure was quantified after 24 h and 48 h for Kyse30 and FaDu cells, respectively. Mean and SD are shown in scatter dot plots of *n* = 3 independent experiments. * ≤ 0.05, ** ≤ 0.01; *** ≤ 0.001, **** ≤ 0.0001. **D** FaDu control (Neg.) and ITGB4_KD cells (KD10 and KD30) spheroids were cultured in Matrigel under serum-free conditions. Spheroids were left untreated (Neg.Ctrl), treated with EGF^high^, or treated with a combination of EGF^high^ and Cetuximab. Representative images of *n* = 3 independent experiments with multiple spheroids are shown. **E** Invasive area representing the outer rim of cells (see yellow lines in D) and invasive distance representing the mean distance covered by 10–15 most invasive single cells were quantified. Mean and SD are shown in scatter dot plots of *n* = 3 independent experiments where each dot represents one spheroid. **** ≤ 0.0001. **F** Representative immunofluorescence confocal images of ITGB4, Ki67, and nuclei in peripheral cells of control- and EGF-treated FaDu spheroids are shown. Merged pictures of the full spheroids are shown in inlays. **G** Examples of tumor budding are shown for HNSCC with homogeneous or edge-localized ITGB4 expression. **H** Proportions of homogeneous and edge localization of ITGB4 in *n* = 106 HNSCC. **I** Budding proportions of HNSCC are depicted for tumors with homogeneous or edge localization of ITGB4 (*n* = 16). **J** Proportions of budding intensities of HNSCC are depicted for tumors with homogeneous or edge localization of ITGB4 (*n* = 16)

ITGB4 expression was knocked down (KD) in Kyse30 and FaDu cells using specific and scrambled shRNA in lentiviral vectors. KD cells generated with different MOI for each cell line were further analyzed (*n* = 2). Wildtype and scrambled shRNA controls (Neg) expressed similar levels of ITGB4, whereas ITGB4-KD resulted in 84% and 87% reduction in Kyse30 cells and in 73% and 68% reduction in FaDu cells (Supplementary Fig. 12). Migration and invasion of ITGB4-KD cells was tested in Boyden chambers in the absence or presence of Matrigel. Treatment of Kyse30 and FaDu cells with EGF^high^ promoted migration and invasion. ITGB4-KD induced a substantial reduction or abrogation of EGF^high^-mediated migration and invasion. Cetuximab inhibited migration and invasion, thus confirming the specificity for EGFR activity (Fig. 7A-B). EGF^high^ treatment also increased wound closure in Kyse30 and FaDu control cells, whereas ITGB4-KD cells migrated only weakly or not at all. Co-treatment of all cell lines with Cetuximab entirely abrogated migration as compared to negative controls (Fig. 7C and Supplementary Fig. 13).

EGFR-mediated local invasion was addressed in a 3D model. Spheroids of FaDu control and ITGB4-KD cells were transferred into Matrigel before addition of EMT-inducing concentrations of EGF (EGF^high^). Treatment of control cells induced a strong invasion into the ECM, which was efficiently inhibited by Cetuximab (Fig. 7D). ITGB4-KD cells showed a reduction of invasion, which was further inhibited by Cetuximab (Fig. 7D). Quantification of invasive area and migratory distance confirmed a strong induction of EGFR-mediated local invasion that was significantly reduced in ITGB4-KD cells. It must further be noted that cell numbers in the invasive area were also considerably diminished as visualized in Fig. 7D. Invasive area and distance were efficiently inhibited by Cetuximab (Fig. 7E). Hence, ITGB4 expression supports EGFR-mediated migration and local invasion.

ITGB4 and the proliferation marker Ki67 were visualized by immunofluorescence staining and confocal imaging in spheroids maintained in Matrigel. Owing to the staining and imaging method, we concentrated on cells of the outer layers to address ITGB4 availability. Control-treated spheroids showed a weak expression of ITGB4, where only the most outer cells expressed higher levels of the protein in areas of contact to Matrigel. Cells at the leading edges of invasion following EGF treatment expressed high levels of ITGB4 and reduced levels of proliferation marker Ki67 (Fig. 7F). Hence, ITGB4 is strongly expressed and available as a target in locally invasive cells.

In primary HNSCC, initial steps of local invasion were assessed as tumor budding, which is defined as single cells or clusters composed of less than 5–10 cells. Budding was observed in primary HNSCC with a homogeneous and an edge localization of ITGB4-positive tumor cells (Fig. 7G). Budding was quantified as negative, weak, intermediate, and strong according to the frequency of tumor buds (Supplementary Fig. 14). Edge localization of ITGB4 was observed in 31.1% of cases (33/106) (Fig. 7H) and was associated with significantly more tumor budding (Fig. 7I) and higher budding intensity than the homogeneous ITGB4 localization (Fig. 7J).

### Antagonizing ITGB4 inhibits local invasion.

**Fig. 8:**
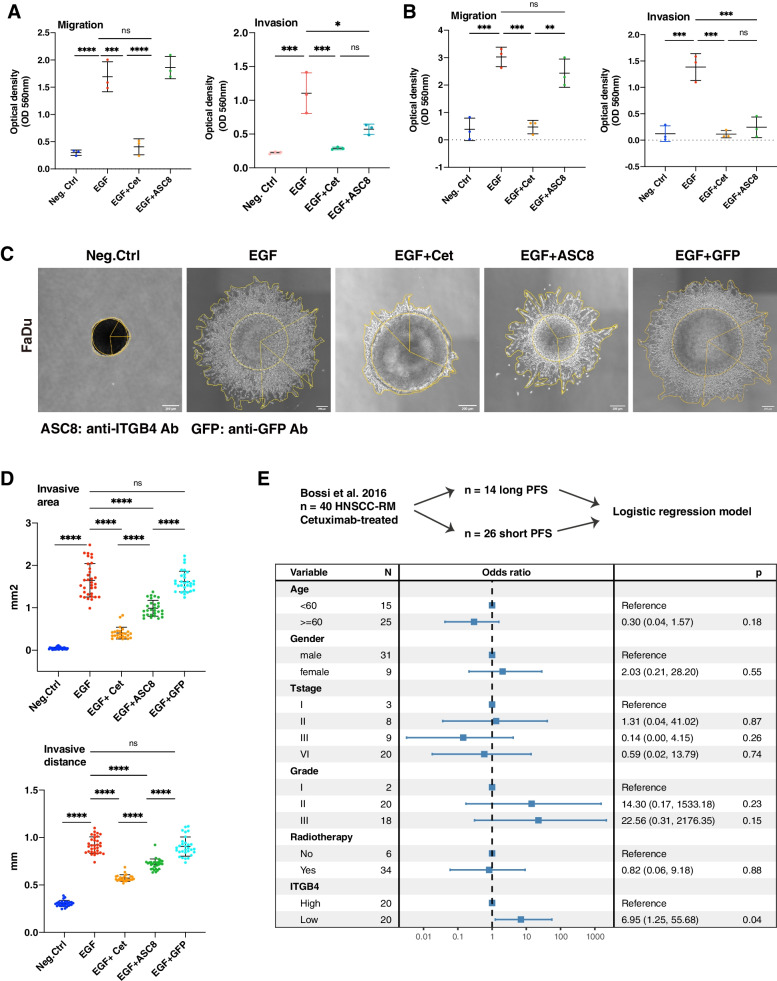
ITGB4 represents a target and potential predictive marker for HNSCC. **A-B** Wildtype Kyse30 and FaDu cells were analyzed in migration assays (**A**) and in invasion assays with inlays coated with Matrigel (**B**) in a Boyden chamber. Where indicated, cells were treated EGF^high^ or combinations of EGF^high^ with Cetuximab or anti-ITGB4 antibody ASC8. Mean and SD are shown in scatter dot plots of *n* = 3 independent experiments. * ≤ 0.05, ** ≤ 0.01; *** ≤ 0.001, **** ≤ 0.0001. **C** Spheroids of wildtype FaDu cells were cultured in Matrigel under serum-free conditions. Spheroids were left untreated (Neg.Ctrl), treated with EGF^high^, or treated with a combination of EGF^high^ and Cetuximab, ASC8, or anti-GFP antibody. Representative images of invasive cells upon treatment (*n* = 3 independent experiments with multiple spheroids) are shown. **D** Invasive area representing the outer rim of cells (see yellow lines in C) and invasive distance representing the mean distance covered by 10–15 most invasive single cells were quantified. Mean and SD are shown in scatter dot plots of *n* = 3 independent experiments where each dot represents one spheroid. **** ≤ 0.0001. **E** Cetuximab-treated recurrent metastatic HNSCC (*n* = 40; GSE65021) were included in a logistic regression analysis. Expression of ITGB4 was stratified at the median. Low ITGB4 expression was associated with higher odds of short PFS (< 5 months; median 3 months; range 1–5) versus long PFS (> 12 months; median 19 months; range 12–36). A Forest plot with event numbers, log-rank p-value, and 95% CI is shown

ITGB4-antagonizing antibody ASC8 was tested in 2D and 3D models. EGF^high^ treatment induced migration and invasion in a Boyden chamber in the absence or presence of Matrigel, which were both blocked upon co-treatment with Cetuximab. Co-treatment with ASC8 antibody had no significant effect on cell migration but blocked invasion comparably to Cetuximab (Fig. 8A-B).

Effects of ASC8 antibody on local invasion were further addressed in spheroids. Wildtype FaDu spheroids embedded in Matrigel were treated with EGF^high^, EGF^high^ plus Cetuximab, EGF^high^ plus ASC8, and EGF^high^ plus an anti-GFP antibody as a control. EGF^high^ treatment induced local invasion of FaDu cells into Matrigel, which was inhibited by Cetuximab treatment. Antagonizing ITGB4 antibody resulted in strong inhibition of invasion, while the control anti-GFP antibody had no effect (Fig. 8C). Quantification of the invasive area (IA) and invasive distance (ID) confirmed a significant and strong increase following treatment with EGF^high^, which was efficiently inhibited by Cetuximab (IA: 76.19% reduction; ID: 37.9%). ASC8 antibody treatment decreased the IA by 40.5% and the ID by 21%, anti-GFP antibody had no significant effect (IA: 6.5%; ID: 2.17%) (Fig. 8D).

### EGFR-mediated EMT and response to Cetuximab

EGFR-mediated EMT may have various repercussions on tumor progression. EMT is generally associated with resistance to Cetuximab [33] and EGFR-mediated EMT promotes tumor invasion. To address potential roles of EGFR-mediated EMT in the regulation of treatment response, unique and overlapping DEGs (|log2FC|> 0.5, *p*-value < 0.05) following Cetuximab treatment in the sensitive HNSCC cell lines SCC1, SCC6 and SCC25 (GSE137524) were determined in the EGFR-mediated EMT signature. Overlapping DEGs were defined as genes with identical directionality of regulation (up- or down-regulated) in SCC cell lines following Cetuximab treatment and the EGFR-mediated EMT signature. A total of 48, 20, and 19 overlapping DEGs with EGFR-mediated EMT (*n* = 171 genes) were determined for SCC1, SCC6, and SCC25, respectively (Supplementary Table 4).

Overlapping DEGs from SCC1, SCC6, and SCC25 were separately assigned to hallmark gene sets of MSigDB using the functions “Investigate Gene Sets” and “Compute Overlaps with Hallmarks”. Overlapping DEGs were significantly enriched in “HALLMARK_E2F-TARGETS”, “HALLMARK_G2M-CHECKPOINT”, and “HALLMARK_MITOTIC_SPINDLE”. All overlapping DEGs contributing to significantly enriched hallmarks involved in cell cycle regulation were down-regulated genes. Intersection of overlapping DEGs from SCC1, SCC6, and SCC25 (MND1, MNS1, CCNB1, HMGB2, PLK1, and CDC20) represents common DEGs between Cetuximab-treated SCC cell lines and the EGFR-mediated EMT. These DEGS confirmed cell cycle down-regulation within “HALLMARK_E2F_TARGETS” and “HALLMARK_G2M_CHECKPOINT”. Hence, genes significantly regulated early upon Cetuximab treatment and in EGFR-mediated EMT suggested a common feature of inhibition of cell cycle progression, which may improve resistance to therapeutic treatment in general and, specifically, upon Cetuximab treatment.

Furthermore, overlapping DEGs between the EGFR-mediated EMT and Cetuximab resistant versus sensitive cells were extracted from the GSE21483 dataset (Cetuximab-sensitive SCC1 cells and their resistant derivative 1Cc8). A total of 22 overlapping DEGs were identified (Supplementary Table 4), however the assignment of these DEGs to MSiGDB gene sets failed to identify significantly enriched hallmarks.

Thus, we conclude that DEGs common to the EGFR-mediated EMT signature and Cetuximab-treated cells are rather related to an early response to treatment and the inhibition of cell cycle progression. Within an established resistance to Cetuximab, DEGs overlapping between EGFR-mediated EMT and Cetuximab resistance are more heterogeneous and did not contribute to any significant enrichment of MSigDB hallmarks.

Next, we concentrated on the potential involvement of genes of the EGFR-mediated EMT signature in functional aspects of tumor progression. As part of a prognostic 5-gene signature of EGFR-mediated EMT, ITGB4 is functional in invasion and its up-regulation is blocked by Cetuximab. Treatment with Cetuximab remains a central tool in multimodal palliative management of progressed metastatic HNSCC, but markers indicative of response to therapy are lacking. Bossi et al*.* (2016) have reported a whole-genome cDNA analysis of a clinical cohort of recurrent-metastatic HNSCC (RM-HNSCC; *n* = 40) that were all treated with chemotherapy in combination with Cetuximab. *N* = 14 patients were characterized by long PFS (median = 19 months) and *n* = 26 patients showed a short PFS (median = 3 months [34]). Assuming ITGB4 might represent a surrogate marker for EGFR-mediated EMT, its association with PFS was assessed. The odd ratio of ITGB4 expression for PFS estimation was adjusted by available demographic and clinical features in a multivariate logistic regression model. Low ITGB4 expression (median cut-off) was significantly associated with higher relative risk of shortened PFS (HR: 6.95, 95% CI 1.25–55.68, *p*-value 0.04) (Fig. 8E). These results suggest that patients with higher ITGB4 expression profited more from Cetuximab treatment.

## Discussion

Molecular analyses of both tumor bulk and single malignant cells provided valuable information on inter- and intratumor heterogeneity of HNSCC and demonstrated the important contribution of EMT and partial forms of it to HNSCC malignant progression [14, 17, 20, 35, 36]. Tumor cells adjacent to the tumor-stroma interface are more prone to an EMT-based trans-differentiation due to interactions with the TME and are conceivably the origin for local invasion and recurrences [14, 17, 20, 35, 36]. A comparison of primary tumors and recurrences demonstrated a prevalence of the basal molecular subtype in recurring tumors and a frequent subtype switch to the basal subtype in recurrences. This subtype was significantly correlated with a pEMT signature and early recurrence of the patients [37]. Thus, peripheral tumor cells in various EMT states are interesting targets to suppress local recurrence and metastases formation, both major sources of clinical complications in HNSCC [8, 38].

The association of EGFR-mediated EMT with progression is of particular interest since Cetuximab is approved to treat locally advanced and recurrent HNSCC [39]. However, no biomarkers for predicting Cetuximab response exist, and, presumably as a lack of predictive biomarkers, treatment efficacy and benefits remain unsatisfying [7]. The present study focused on transcriptomic changes related to EGFR-mediated EMT and opportunities to define prognostic and therapeutic targets. We show that different concentrations of EGF and the EpCAM-derived EGFR ligand EpEX initially induce a significant enrichment of the “EMT” MSigDB gene set in a GSEA. However, only a sustained strong activation with high-dose EGF resulted in morphologic and functional features of EMT and in high numbers of DEGs (Fig. 2). These results are in line with earlier reports on the duality in signaling outcome of EGFR towards either proliferation or EMT depending on ERK1/2 activation strength. Sustained strong activation was required for EMT *in vitro* [21]. Similar findings were reported for other carcinoma entities such as breast cancer, where a sustained intermediate activation or a strong short-term induction *in vitro* promoted proliferation, while sustained strong ERK activation suppressed proliferation and induced EMT [40].

EpEX was reported as an EGFR ligand in colorectal and HNSCC cancer cells and in mesenchymal stem cells [21, 23–25]. We show at the transcriptional level that EpEX is a weak ligand of EGFR that induces fewer target genes than EGF and that shared DEGs of EpEX and EGF were identically regulated in vitro. Accordingly, EpEX-dependent inhibition of EGFR-mediated EMT does not rely on transcriptional repression but on a competition with EGF for binding to EGFR (Fig. 3). As a net effect EpEX reduces ERK1/2 induction and prevents EMT [21].

The EGFR-mediated EMT signature (n = 171) reflected molecular changes in cell–matrix interaction, cell–cell adhesion, and the formation of cell leading edges, at the expense of proliferation. These shifts are classical hallmarks of EMT that are associated with improved migration, invasion, and resistance to drugs used in systemic therapy [12, 41]. EGFR-mediated EMT defined cells in a state of EMT comparably with the MSigDB EMT hallmark and the pEMT [10] gene signatures but showed differences regarding its enrichment at the single cell level. This finding is corroborated by low numbers of overlapping genes with the EMT hallmark and pEMT signatures (Supplementary Fig. 4). We therefore suggest that EGFR-mediated EMT represents a distinct meta-program of EMT that deserves further investigation in future studies.

Assuming a detrimental effect of EGFR-mediated EMT on clinical outcome, signature genes associated with poor OS were selected. Forward feature selection and multivariate Cox proportional hazard regression models defined a 5-gene signature composed of DDIT4, FADD, ITGB4, NCEH1, and TIMP1 that prognosticated OS in the TCGA-HNSCC cohort. The risk score was re-assessed in validation cohorts by transferring weighing factors and median cut-off threshold from the TCGA cohort. Both validation cohorts showed a significant 5-gene signature-based split and balanced distributions of patients in both strata, thus confirming the association of the 5-gene signature with OS (Fig. 4). We further compared the EGFR-mediated EMT-based risk score with published EMT signatures including the MSigDB EMT hallmark, the pEMT [10], and two EMT signatures [29, 30] regarding their prognostic value to predict OS. EGFR-mediated EMT- and pEMT-based risk scores were superior to the three EMT-based risk scores, suggesting a more refined classification of patients (Supplementary Fig. 5).

Correlation of all five genes with PROGENy pathway activities demonstrated an association of ITGB4 with EGFR and MAPK activity, which are both required for EGFR-mediated EMT [21]. ITGB4 correlation with EGFR activity was confirmed in malignant single cells and over-expression in primary tumors and metastases was independent of the HPV-status (Fig. 5). This finding is in accordance with mRNA and protein expression of ITGB4 in a large cohort of *n* = 2330 HNSCC patients, in which ITGB4 was upregulated in HNSCC and distinguished HNSCC from non-HNSCC tissue [42]. Co-localization of ITGB4 and its ligand laminin 5 was observed at the tumor-stroma interface and an absence of ITGB4 expression correlated with a lack of laminin 5 deposition, demonstrating their co-dependence (Fig. 5). All components of this receptor-ligand complex, *i.e*. ITGA6, ITGB4, LAMA3, LAMB3, and LAMC2, were up-regulated in HNSCC mRNA expression data (Supplementary Figs. 4– 8), and significantly correlated to ITGB4 in bulk tumor and in malignant single cells mRNA expression data (Supplementary Fig. 3). LAMA3, LAMB3, and LAMC2 were among the top ten genes most strongly correlated to ITGB4 expression (Supplementary Table 2) and are part of the common pEMT signature defined by Puram et al*.* [10]. A survey of scRNA-seq datasets from *n* = 28 different cancer entities further demonstrated that ITGB4 showed the strongest and most strongly tumor-associated expression in HNSCC compared to all other tumors.

Involvement of ITGB4 in migration/invasion, stemness, and EMT is documented for several carcinomas. In triple-negative breast cancer, ITGB4 marks cancer stem cells in pEMT and was associated with reduced relapse-free survival following chemotherapy [43]. Targeting ITGB4 in mouse models of breast cancer and HNSCC with dendritic cells pulsed with ITGB4 protein or with a CD3/ITGB4 bispecific antibody inhibited tumor growth and metastasis formation [44]. Hence, ITGB4 is recognized as prognostic marker with mechanistic implication in tumor progression of breast cancer. In HNSCC, we show that ITGB4 is crucial to EGFR-mediated migration and local invasion both in 2D and 3D models. In situ immunostaining of ITGB4 and the proliferation marker Ki67 in invasively growing spheroids demonstrated a strong ITGB4 expression in early invading cells in conjunction with reduced Ki67 expression. These results are in line with general features of EMT and with the enrichment of cellular components of focal adhesion and cell leading edge, and the suppression of cell cycle and DNA replication in the EGFR-mediated EMT signature. Additionally, strong expression of ITGB4 in leading invasive cells as shown in this study confirmed its suitability for therapeutic targeting. *In vivo*, preferential ITGB4 expression in peripheral cells of tumor areas was significantly associated with a higher degree of tumor budding, which is a hallmark of local invasion and metastasis formation associated with reduced OS [45]. In this study, ITGB4 was expressed in tumor protrusions and in detached tumor buds, further supporting a possibility of targeting ITGB4 to impede early steps of local invasion and metastasis formation. As proof-of-concept for this therapeutic approach, antagonizing ITGB4´s interaction with laminin 5 with the ASC8 antibody strongly reduced tumor cell invasion, confirming the potential of ITGB4 as therapeutic target to block local invasion. Induction of EMT in tumor cells is a central therapy resistance mechanism to receptor tyrosine kinase treatment including Cetuximab [46]. ITGB4-targeted therapy might represent a resort to Cetuximab resistance using either antagonizing antibodies as presented here or immunomodulatory approaches including ITGB4-primed dendritic cells or bispecific antibodies targeting T cells and ITGB4-positive tumor cells [44].

Under physiological conditions, ITGB4 and laminin 5 are essential in anchoring epithelial cells at the basal membrane. In tumor progression however, ITGB4 and laminin 5 are involved in local invasion, thus fostering tumor cell dissemination and the generation of a minimal residual disease composed of occult tumor cells that escape classical imaging technologies [47]. Here, laminin 5 acts as a structural ligand that generates a scaffold for the binding of ITGB4, thereby leading the way for tumor migration and invasion [48]. Moreover, laminin 5 triggers integrin-dependent intracellular signaling including the PI3K pathway, which increases migration, invasion, and EMT [49]. Here, the subunit LAMC2 has a special function that arises from its changed cleavage products in carcinoma. N-terminally located EGF repeats present in LAMC2 are cleaved by MMP-2 or MT1-MMP and activate EGFR and the MAPK pathway to foster migration and survival of cancer cells [49]. Hence, the up-regulation of LAMC2 in HNSCC cells that have transited to a pEMT state upon EGFR activation may represent a positive feedback loop that enforces EGFR effects. Furthermore, ITGB4 cooperates with EGFR to foster resistance to anoikis in hepatocellular carcinoma [50]. Anoikis is an important aspect of tumor progression that confers enhanced independence from cell–cell contact and anchorage during the initial detachment of carcinoma cells from the primary tumor. Lastly, the coordinated formation of a pseudo-basement membrane surrounding tumor areas acts as a physical barrier for therapeutic drugs and non-malignant cells, including immune cells [51].

Patients with a high degree of EGFR-mediated EMT and an associated propensity to develop local recurrences or metastases will supposedly profit most strongly from Cetuximab treatment. Analysis of Cetuximab-treated HNSCC patients suffering from recurrent metastatic disease [34] showed that patients with low ITGB4 expression were at higher relative risk to have short PFS. These findings point towards a higher benefit of Cetuximab treatment for RM-HNSCC patients with higher expression levels of ITGB4, as surrogate for enhanced EGFR-mediated EMT. Thus, initial results support a potential role for high ITGB4 expression as predictive marker for Cetuximab treatment, which requires further investigation in prospective studies.

## Conclusions

A transcriptomic map of EGFR-mediated EMT was established that allowed defining a prognostic risk score for HNSCC patients. In the frame of EGFR-mediated EMT, ITGB4 was identified as a mechanistic biomarker that is essential for local invasion, is associated with tumor budding, is a target for antagonizing antibodies, and represents a novel predictive marker candidate. Both, EGFR signaling and EMT are central regulators of tumor progression in HNSCC. Therefore, the present study is an important contribution to the understanding of molecular mechanisms involved in HNSCC progression with potential implications in EGFR-based treatment.

## Methods

### Cell lines and treatments

Kyse30 and FaDu (HPV-neg.) (ATCC, Manassas, VA, USA) were regularly confirmed via STR typing. Cells were passaged in DMEM or RPMI, 10% FCS, 1% penicillin/streptomycin, 5% CO_2_ atmosphere at 37 °C. Treatment with EGF (1.8–9 nM, PromoCell PromoKine, Heidelberg, Germany), EpEX-Fc [21] (10-50n nM), Cetuximab (10 µg/mL, Erbitux, Merck Serono, Darmstadt, Germany), anti-ITGB4 (10 µg/mL, ASC8, Merck, Germany), anti-GFP antibody (10 µg/mL, Thermo Fisher Scientific, Germany), AZD6244 (1 µM, Selleckchem, Munich, Germany), MK2206 (1 µM; Selleckchem, Munich, Germany) were conducted under serum-free conditions. Recombinant immunoglobulin Fc region (Fc) served as control EpEX-Fc (Jackson ImmunoResearch, Baltimore, US).

Stable knock-down of ITGB4 in Kyse30 and FaDu cells was done by lentiviral transfer [52] of an shRNA targeting ITGB4 (shITGB4: 5’-CGAGAAGCTTCACACCTAT-3’). As negative control, an irrelevant scrambled shRNA (shControl: 5 ‘-CCTAAGGTTAAGTCGCCCT-3’) was used. The lentiviral backbone pLVX-shRNA1 (Clontech, Saint-Germain-en-Laye, France) was packaged in 293 T cells using the plasmids psPAX2 and phCMV-VSV-G. For transduction, 50.000 cells were plated per well of a 24-well plate in 500 µL medium and 10 µL or 30 µL of non-concentrated supernatant containing lentiviral particles were added, respectively. Besides the shRNA, the vector also transferred a resistance against puromycin, which was used to select for stable ITGB4-KD cells by application of 1 µg/mL puromycin. KD of ITGB4 was confirmed by flow cytometry.

### Flow cytometry

ITGB4 was stained with antibody 439-9B (Thermo Fisher Scientific, Germany, 1:200 in PBS-3% FCS, 60 min on ice), cells were washed three times in PBS-3% FCS and stained with FITC-conjugated secondary antibody (Vector Laboratories/Biozol, Eching, Germany; FI-4001; 1:50; 45 min). Fluorescence intensity was assessed in a CytoFlex using CytExpert Software, Version 2.2 (Beckman Coulter, Krefeld, Germany) and the FlowJo software version 10.8.1 (FlowJo, Ashland, OR, USA).

### Immunohistochemistry, immunofluorescence, and scoring

ITGB4 (439-9B, 1:200, Thermo Fisher Scientific, Germany) and laminin 5 (P3H9, 1:500, Abcam, Germany) antibodies were used for immunohistochemical and immunofluorescence staining in combination with avidin–biotin-peroxidase method (Vectastain, Vector laboratories, Burlingame, CA, US) or Alexa Fluor-488- and Alexa Fluor-594-conjugated secondary antibodies. Confocal microscopy imaging was conducted with a TCS-SP8 scanning system and a DM-IRB inverted microscope (Leica, Nussloch, Germany). Immunohistochemical scoring was quantified by two experienced scorers blinded for sample identities as described before [53].

### Reverse transcription qPCR

Total RNA was extracted with the RNeasy Mini Kit (Qiagen, Germany) and reverse-transcribed with the QuantiTect Reverse Transcription kit (Qiagen, Germany). cDNAs were quantified in triplicates by qRT-PCR using SYBR-Green Master PCR mix with gene-specific primers in a QuantStudio3 device (Thermo Fisher Scientific, Germany). All mRNA quantifications were normalized to TBP. The following primers were used:

- TBP-FW 5’-CCA CTC ACA GAC TCT CAC AAC-3’
- TBP-BW 5’-CTG CGG TAC AAT CCC AGA ACT-3’
- ITGB4-FW 5’-CTC CAC CGA GTC AGC CTT C-3’
- ITGB4-BW 5’-CGG GTA GTC CTG TGT CCT GTA-3’

### 2D migration and invasion

Migration and invasion assays were conducted as described [14] in transwell chambers (8.0 µm, Merck Millipore Ltd., Germany) without or with Matrigel coating (1 mg/ml, Corning, Germany), respectively. Cells at density of 2.5 × 10^5^ in 300 µl serum-free medium were seeded into upper inserts and different treatment media were placed in the lower chamber. After 24 h, migrated and invaded cells were quantified with the QCM™ 24-Well Colorimetric Cell Migration Assay Kit (Merck Millipore Ltd., Germany) in a colorimeter (VersaMax Microplate Reader, Molecular Devices, San Jose, CA, USA).

### Wound healing assay

Kyse30 and FaDu were cultured to full confluence under serum-free conditions. Cell layers were scratched with a sterile 200 µL pipette tip. Quantification of scratches was performed at indicated time points using ImageJ and MRI wound healing tool (NIH, Bethesda, MD, USA).

### 3D invasion

FaDu cells (3000 cells/well) were seeded in 96-well low-adherent plates and spheroids formed for 72 h. Glass bottom dishes (35 mm, Ibidi, Germany) were coated with 40 µL of 3 mg/mL Matrigel (Corning, Germany), spheroids embedded in 160 µL of 3 mg/mL Matrigel and plated onto coated dishes (37 °C, 1 h). After Matrigel polymerization, dishes were filled with medium containing indicated treatments. After 72 h, invasive cells were imaged (Leica, Nussloch, Germany). Invasive area (*i.e.* cell-covered area except the area of the original spheroid) and invasive distance (*i.e.* distance of the 10–15 cells furthest from the center of the spheroid) were quantified by Image J.

### EGF competition assay

Kyse30 and FaDu cells (5 × 10^5^ cells/well in 6-well plates) were incubated with Alexa-488-conjugated EGF (18 nM, ThermoFisher, Munich, Germany) alone or in combination with unlabeled EGF (18 nM, PromoKine, Heidelberg, Germany) or unlabeled EpEX-Fc (18 nM) for 1 h. Labeled cells were washed three times in FACS buffer (PBS; 3% FCS) before analysis in a CytoFlex (Beckman Coulter, Krefeld, Germany).

### Cell line RNA sequencing

RNA was extracted using the RNeasy Mini Kit (Qiagen, Germany) according to the manufacturer’s protocol. Prior to library generation, RNA was quantified using the Qubit™ RNA BR Assay Kit (#Q10210) with the Qubit Fluorometer (Thermo Fisher Scientific, MA, USA). RNA integrity was assessed determining DV200 values (percentage of fragments > 200 nucleotides) using the 2100 Bioanalyzer (Agilent Technologies, Inc., USA) in combination with the Agilent RNA 6000 Nano Kit (#5067–1511). RNA sequencing libraries were prepared with 100 ng input of total RNA using the QuantSeq 3′ mRNA-Seq Library Prep Kit FWD for Illumina (#SKU:015.96; Lexogen, Austria) following the manufacturer’s instructions for single indexing and good RNA quality. For library amplification, PCR cycles were determined with the PCR Add-on Kit for Illumina (#SKU:020.96, Lexogen) and the individual libraries were amplified with 19 or 20 PCR cycles. Quality and quantity of the libraries were evaluated using the Quanti-iT PicoGreen dsDNA Assay Kit (P7589, Thermo Fisher) and the Bioanalyzer High Sensitivity DNA Analysis Kit (#5067–4626, Agilent Technologies). Sequencing of libraries was performed with 150 bp paired-end mode.

### Clinical RNA expression datasets

Normalized RNA expression from the TCGA-HNSCC cohort [5] and clinical data were downloaded from cBioPortal (https://www.cbioportal.org/) with the CGDS package. RNA count expression data were downloaded from *Xena* (https://xenabrowser.net/). Differential gene expression was compared by log2(CPM + 1). Microarray-based HPV- HNSCC cohorts from MD Anderson Cancer Centre (MDACC) and Fred Hutchinson Cancer Research Center (FHCRC) were retrieved from Gene Expression Omnibus (GEO; GSE42743, GSE41613). All RNA expression values were log2 transformed and scaled before training and validating prognostic Cox regression models. Only HPV-negative cases were included resulting in total sample sizes of *n* = 240 (TCGA), *n* = 74 (MDACC), and *n* = 97 (FHCRC). Further details are compiled in Supplementary Table 2. Transcriptomic data of Cetuximab-treated patients (*n* = 40) and cetuximab-resistant and -sensitive cell lines were downloaded from GEO (GSE65021, GSE21483). Pre-processed HNSCC single-cell dataset was downloaded from GEO (GSE103322). As described by Puram et al*.* [10], ten samples containing most malignant cells were included into further analysis. The additional siRNA-seq dataset consisting of control- and Cetuximab-treated HNSCC cell lines was downloaded from GEO (GSE137524). All scRNA-seq datasets were imported as Seurat objects and analyzed using *Seurat R* (AddModuleScore) and Gene Set Variation Analysis (GSVA).

### Differential expression (DE) analysis and functional enrichment analysis

DE analysis for microarray data, bulk RNA-seq, and scRNA-seq data were conducted by using *Limma*, *DEseq2*, and *Seurat* packages in R, respectively. |log2FC|> 0.5 and adjusted p-value ≤ 0.05 were used as the threshold for differentially expressed genes (DEGs). For matched samples of normal mucosa and HNSCC in TCGA-HNSCC, genes with top 25% expression were adopted for principal component analysis (PCA) and heatmap plots indicating variance between normal and tumor samples. The geom_mark_ellipse function (*ggforce)* was implemented for area annotation in PCA plots. Venn diagrams and UpSet plot were utilized to visualize common and exclusive genes across comparisons. Gene set enrichment analysis (GSEA) was performed with genes ranked by fold-change of DE or correlation between genes using hallmark gene sets in the Molecular Signatures Database (MSigDB), Gene Ontology terms (GO), and Kyoto Encyclopedia of Genes and Genomes (KEGG). Over representation analysis (ORA) was applied for functional enrichment analysis of the EGFR-mediated EMT signature. The enrichment analysis was conducted and visualized with *clusterProfiler* package and *enrichplot* package (Bioconductor), respectively. Bulk-seq samples and malignant cells from scRNA-seq datasets were uniformly scored by GSVA to quantify different EMT programs using the pEMT, MSigDB EMT hallmark, and EGFR-mediated EMT signatures. Additionally, the pEMT score of malignant single cells was calculated with the AddModuleScore function (*Seurat*), which was processed in accordance with the original report [10].

### Survival analysis

Overall survival was the main clinical endpoint in the present study. Univariable Cox regression models were employed for screening genes in the EGFR-mediated EMT gene set. Up-regulated genes with a hazard ratio (HR) > 1 or down-regulated with a HR < 1 were analyzed further. *Rbsurv* package (Bioconductor) was used for establishing multivariate Cox regression model based on the TCGA-HNSCC cohort. Risk scores were calculated by summing up the products of scaled gene expression and respective coefficient. Median value of risk score in TCGA cohort was applied for prognostic stratification and was then transferred to dichotomize validation cohorts. Kaplan–Meier curves were utilized to visualize survival differences of clinical cohorts.

Four EMT-related signatures served to compare the prognostic prediction capacity of the EGFR-mediated EMT signature (MSigDB hallmark EMT, pEMT by Puram et al*.* [10], and EMT signatures by Jung et al. [29] and Vallina et al*.* [30]). Feature selection with *Rbsurv* and multivariate Cox regression models were establishment in HPV-negative patients of the TCGA-HNSCC cohort [5] using the abovementioned EMT-related signatures. 3-year and 5-year OS predictions of all models were visualized as receiver operating characteristic (ROC) curves with denoted area under curve (AUC).

### Pathway activation inference

Based on tumor mRNA expression data, the signaling activity of 14 pathways was computed by Pathway RespOnse GENes for activity inference (*PROGENy*) [31]. Correlation and significance between genes and pathway activation were visualized with the *corrplot* R function.

### Statistical analyses

The expression differences between sample groups were compared by t-test and one-way ANOVA. Correlation between gene expression was calculated by Spearman’s correlation. A multivariate logistic regression model was applied for testing the odds ratio of gene expression level with relapse period since anti-EGFR treatment. Data analyses were performed in R (version 4.1.2 (2021–11-01)).

### Tissue samples

Tumor tissue in the LMU cohort was taken by 8 mm punch biopsies of macroscopically vital areas of the primary carcinoma after resection. Normal mucosa samples derived from macroscopically healthy mucosa beyond resection margins that were deemed free from tumor invasion or dysplastic lesions by intraoperative frozen section analyses. Tissue samples were covered with Ringer solution and were immediately dried and embedded in Tissue Tek medium for snap freezing in liquid nitrogen. Demographic and clinical parameters are compiled in Supplementary Table 1.

### Cancer cell line encyclopedia

Data from the Cancer Cell Line Encyclopedia (CCLE) was downloaded from the Broad Institute (“https://data.broadinstitute.org/ccle/”). Cell line data from “UPPER_AERODIGESTIVE_TRACT” and “OESOPHAGUS” was extracted. For CNV data, *n* = 114 cell lines were available; for expression data *n* = 88 cell lines were available. Data were further processed in R.

### Tumor budding analysis

Tumor budding was defined as single tumor cells or clusters of less than five cells detached from main tumor areas [54]. Budding intensity was assessed by two experienced scientists/pathologists blinded to clinicopathologic data and was categorized as negative (no budding visible), weak, intermediate, and strong.

## Supplementary Information

**Additional file 1: SupplementaryFigure 1.** Copy number variation and expression of EGFR in Kyse30and FaDu cells.** Supplementary Figure 2.** GSEA of EGF- and EpEX-treated Kyse30and FaDu cells. **Supplementary Figure 3.** Over-representation analysis of genesof the EGFR-mediated EMT signature.** SupplementaryFigure 4.** Comparison ofEGFR-mediated EMT, pEMT, and EMT signatures.** Supplementary Figure 5.** Comparisonof EMT signatures for prognostic purposes.**Supplementary Figure 6.** ITGB4,ITGA6, LAMA3, LAMB3, and LAMC2 expression in HNSCC.** Supplementary Figure 7.** ITGB4expression in malignant and non-malignant single cells in different cancerentities.** Supplementary Figure 8.** ITGA6 expression in malignant andnon-malignant single cells in different cancer entities.** Supplementary Figure 9.** LAMA3expression in malignant and non-malignant single cells in different cancerentities.** Supplementary Figure 10.** LAMB3 expression in malignant andnon-malignant single cells in different cancer entities.** Supplementary Figure 11.** LAMC2expression in malignant and non-malignant single cells in different cancerentities.** Supplementary Figure 12.** ITGB4 expression in knockdown clonesof Kyse30 and FaDu cells. **Supplementary Figure 13.** Wound healing capacity of control andITGB4-knockdown cell lines.** SupplementaryFigure 14.** Tumor buddingintensities in HNSCC.

**Additional file 2: Supplementary Table 1.** EGFR-mediated EMT.

**Additional file 3: Supplementary Table 2.** TCGA HPV- HNSC cohort.

**Additional file 4: Supplementary Table 3.** Gene expression correlation with ITGB4 in the HPV-negativeTCGA cohort. Batch correlation analysis identified correlations of geneexpression with integrin beta 4 (ITGB4). Gene ID, Spearman correlation, andp-value are indicated for the top ten positively (co-regulated) and negativelycorrelated genes (counterregulated).

**Additional file 5: Supplementary Table 4.** SCC1 cell line: DEGs overlapping with EGFR-mediated EMT signature.

## Data Availability

Datasets generated during and/or analyzed during the current study are either publicly available (TCGA [5], MDACC (GEO object GSE42743) and FHCRC (GEO object GSE41613) or are deposited at Gene Expression Omnibus (GEO) under GSE200421. All codes and R-packages used in the study are publicly available and have been disclosed in Methods or are available from the corresponding authors on reasonable request.

## Abbreviations

CCLE: Cancer Cell Lines Encyclopedia
CNV: Copy Number Variation
DDIT4: DNA Damage Inducible Transcript 4
DEG: Differentially Expressed Gene
EGF: Epidermal Growth Factor
EGFR: Epidermal Growth Factor Receptor
EMT: Epithelial-to-Mesenchymal Transition
EpCAM: Epithelial Cell Adhesion Molecule
EpEX: EpCAM Extracellular domain
FADD: Fas-Associated Death Domain protein
Fc: Fragment crystallizable
FHCRC: Fred Hutchinson Cancer Research Center
GSEA: Gene Set Enrichment Analysis
GSVA: Gene Set Variation Analysis
GO: Gene Ontology
HNSCC: Head and Neck Squamous Cell Carcinoma
HPV: Human Papilloma Virus
HR: Hazard Ratio
ITGA6: Integrin Alpha 6
ITGB1: Integrin Beta 1
ITGB4: Integrin Beta 4
KD: Knock-Down
KEGG: Kyoto Encyclopedia of Genes and Genomes
LAMA3: Laminin Alpha 3
LAMB3: Laminin Beta 3
LAMC2: Laminin Gamma 2
|log2FC|: Log2 Fold Change
MAPK: Mitogen-Activated Protein Kinase
MDACC: MD Anderson Cancer Center
NCEH1: Neutral Cholesterol Ester Hydrolase 1
ORA: Overrepresentation Analysis
OS: Overall Survival
pEMT: Partial EMT
PFS: Progression-Free Survival
PI3K: Phosphatidylinositol-4,5-Bisphosphate 3-Kinase
qRT-PCR: Quantitative Reverse Transcriptase Polymerase Chain Reaction
RNA-seq: RNA sequencing
scRNA-seq: Single cell RNA-seq
shRNA: Short hairpin RNA
TCGA: The Cancer Genome Atlas
TGFβ: Transforming Growth Factor beta
TGFβ-R: Transforming Growth Factor beta Receptor
TIMP1: TIMP Metallopeptidase Inhibitor 1
