# Supplementary material for: A transcriptomic map of EGFR-induced epithelial-to-mesenchymal transition identifies prognostic and therapeutic targets for head and neck cancer
Source: Mol Cancer. 2022 Sep 8;21:178. doi: 10.1186/s12943-022-01646-1 (PMC9454230; doi:10.1186/s12943-022-01646-1)
Supplement: Supplementary file 1 — Additional file 1: SupplementaryFigure 1. Copy number variation and expression of EGFR in Kyse30and FaDu cells. Supplementary Figure 2. GSEA of EGF- and EpEX-treated Kyse30and FaDu cells. Supplementary Figure 3. Over-representation analysis of genesof the EGFR-mediated EMT signature. SupplementaryFigure 4. Comparison ofEGFR-mediated EMT, pEMT, and EMT signatures. Supplementary Figure 5. Comparisonof EMT signatures for prognostic purposes.Supplementary Figure 6. ITGB4,ITGA6, LAMA3, LAMB3, and LAMC2 expression in HNSCC. Supplementary Figure 7. ITGB4expression in malignant and non-malignant single cells in different cancerentities. Supplementary Figure 8. ITGA6 expression in malignant andnon-malignant single cells in different cancer entities. Supplementary Figure 9. LAMA3expression in malignant and non-malignant single cells in different cancerentities. Supplementary Figure 10. LAMB3 expression in malignant andnon-malignant single cells in different cancer entities. Supplementary Figure 11. LAMC2expression in malignant and non-malignant single cells in different cancerentities. Supplementary Figure 12. ITGB4 expression in knockdown clonesof Kyse30 and FaDu cells. Supplementary Figure 13. Wound healing capacity of control andITGB4-knockdown cell lines. SupplementaryFigure 14. Tumor buddingintensities in HNSCC. [file 12943_2022_1646_MOESM1_ESM.docx]

**Supplementary Figures to:**

**A Transcriptomic Map of EGFR-induced Epithelial-to-Mesenchymal Transition Identifies Prognostic and Therapeutic Targets for Head and Neck Cancer**

*Henrik Schinke^1,*^, Enxian Shi^1,*^, Zhongyang Lin^1,*^, Tanja Quadt^1^, Gisela Kranz^1^, Jiefu Zhou^1^, Julia Hess^2^, Claus Belka^3,4^, Horst Zitzelsberger^,4^, Udo Schumacher^5^, Sandra Genduso^5^, Kristoffer Riecken^6^, Yujing Gao^1^, Zhengquan Wu^1^, Christoph Walz^7^, Martin Canis^1^, Kristian Unger^2,3^, Philipp Baumeister^1,2^, Min Pan^1,8^, and Olivier Gires ^1,2^*

**Supplementary Figure 1:** **Copy number variation and expression of EGFR in Kyse30 and FaDu cells.** Copy numbers (**A**) and gene expression (**B**) of EGFR was assessed in cell lines of the upper aerodigestive tract including esophageal and HNSCC cell lines from the Cancer Cell Line Encyclopedia (CCLE).

**Supplementary Figure 2:** **GSEA of EGF- and EpEX-treated Kyse30 and FaDu cells.** (**A-B**) Gene set enrichment analyses (GSEA) were performed for Kyse30 and FaDu cells at 6h and 72h after the indicated treatment (EGF^low^: EGFL, EGF^high^: EGFH, EpEX, EGF^high^ with EpEX: EGF_w_EpEX) using (A) the Gene Ontology (GO) and (B) the Kyoto Encyclopedia of Genes and Genomes (KEGG) terms. Significantly activated or suppressed terms are depicted with gene ratios and adjusted p-values for Kyse30 and FaDu cells at 6h and 72h of the respective activation.


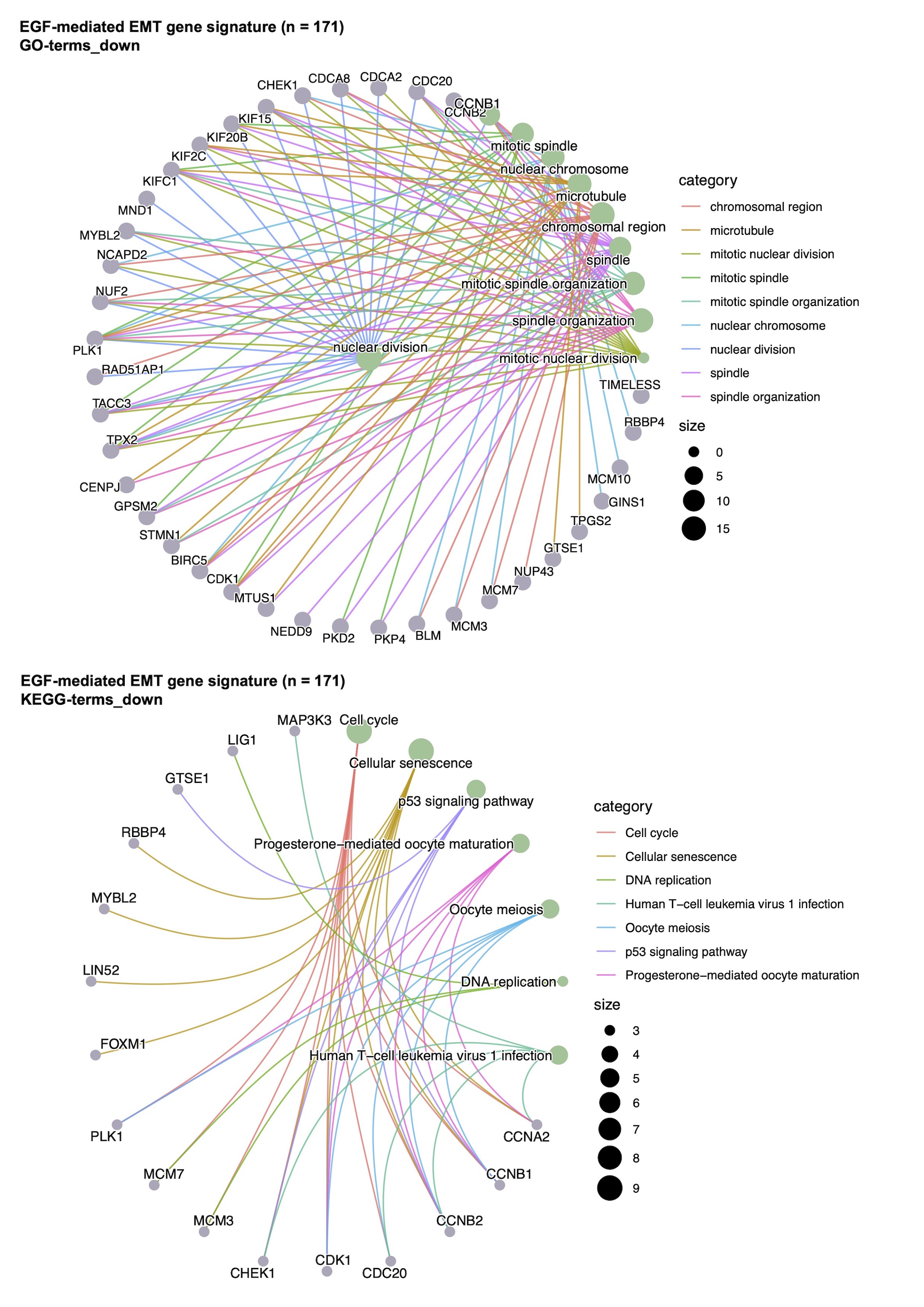


**Supplementary Figure 3:** **Over-representation analysis of genes of the EGFR-mediated EMT signature.** All genes of the EGFR-mediated EMT signature (n = 171) were subjected to an over-representation analysis (ORA). Significantly suppressed pathways in GO and KEGG are depicted in a gene-concept network including the gene numbers for each category. Terms/Categories are represented in green circles, genes are represented in grey circles.


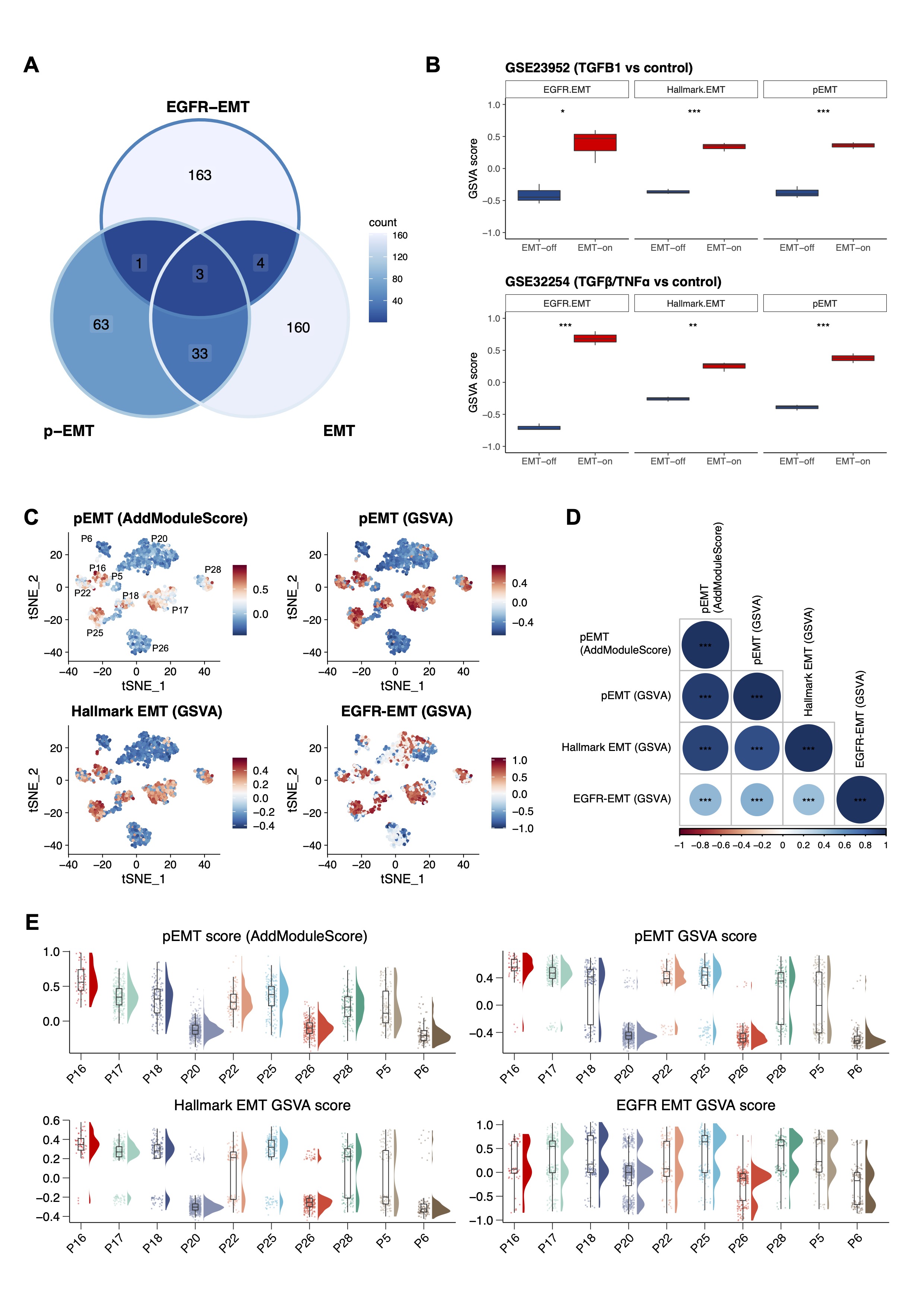


**Supplementary Figure 4:** **Comparison of EGFR-mediated EMT, pEMT, and EMT signatures.** (**A**) Venn diagram of unique and overlapping genes of all three EMT signatures. (**B**) GSVA scores for EGFR-mediated EMT, EMT, and pEMT were calculated in the refinement GSE23952 dataset and in the validation GSE32254 dataset of the MSigDB EMT hallmark. Results are displayed as boxplot whiskers for uninduced cells (EMT-off) and induced cells (EMT-on). Mann-Whitney p-value * < 0.05; ** < 0.01; *** < 0.001. (**C**) GSVA scores for EGFR-mediated EMT, EMT, and pEMT were calculated for malignant cells in the GSE103322 scRNA-seq dataset. Additionally, pEMT scores were re-calculated using AddModuleScore (Seurat R) in analogy to Puram *et al*. All scores for n = 2,176 single malignant cells were depicted as t-SNE plots. (**D**) Correlation plot of AddModuleScore and GSVA scores for pEMT, EMT, and EGFR-mediated EMT signatures in the GSE103322 dataset. *** p-value < 0.0001. (**E**) Boxplot whiskers/Violin plots of AddModuleScore and GSVA scores for pEMT, EMT, and EGFR-mediated EMT signatures in the GSE103322 dataset for each individual patient with median (n = 10).

**(Next page) Supplementary Figure 5:** **Comparison of EMT signatures for prognostic purposes.** Feature selection and multivariate Cox models were computed for the MSigDB EMT hallmark signature (**A**), the pEMT HNSCC signature (Puram et al.) (**B**), and the EMT HNSCC signatures by Jung *et al.* (**C**) and Vallina *et al.* (**D**). Left panels: Shown are Forest plots of the multivariable Cox PH regression model with the incorporating genes of each risk score in the TCGA cohort (n = 240 HPV-negative HNSCC) with event numbers, log-rank p-value, AIC, and concordance index. Right panels: Stratification of HPV-negative HNSCC (TCGA; n = 240) with the indicated risk scores (median cut-off) for overall survival (time in months). Numbers at risk, HR, 95% CI, and p-value are indicated in the Kaplan-Meier survival curve.

**
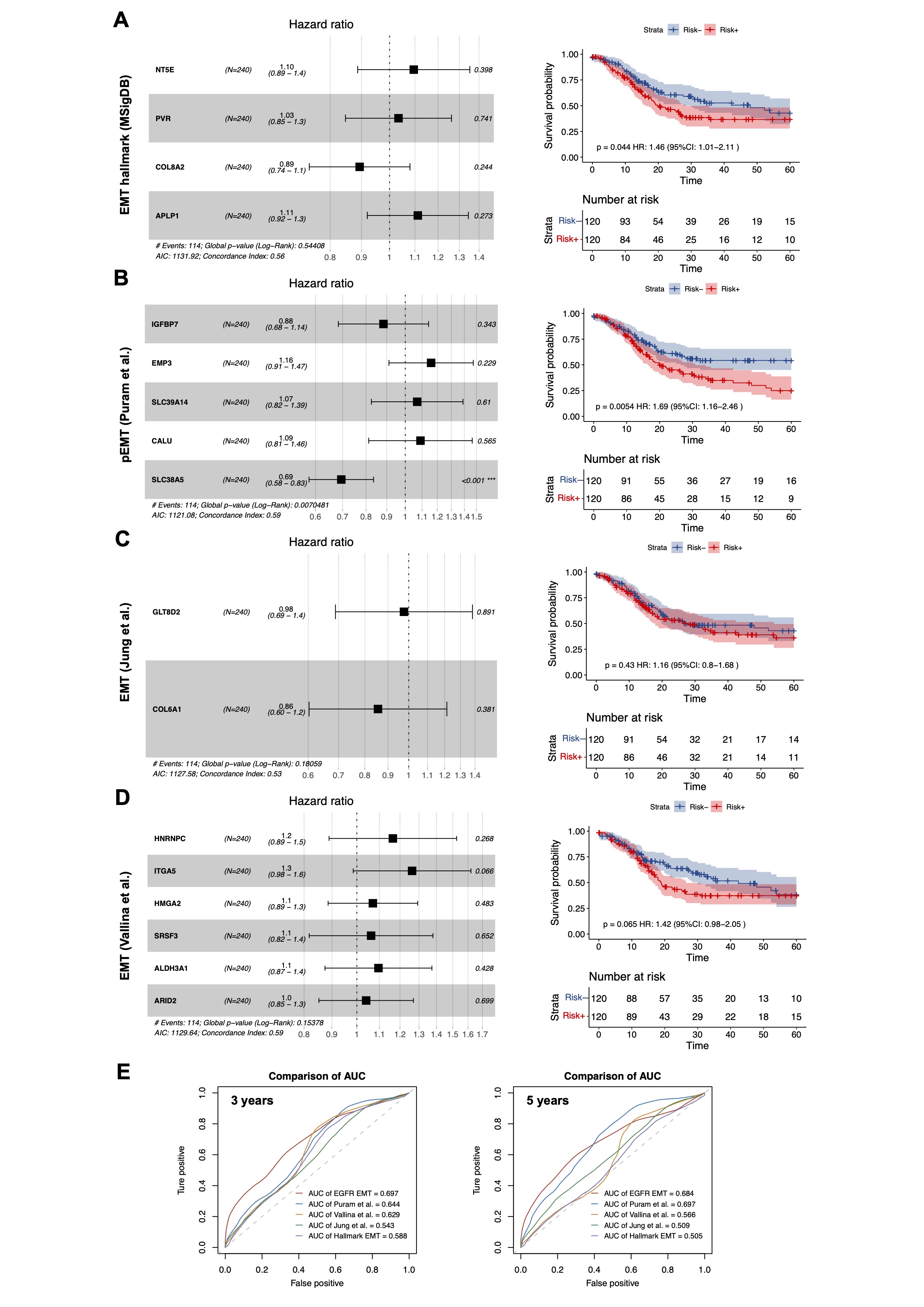
**

**
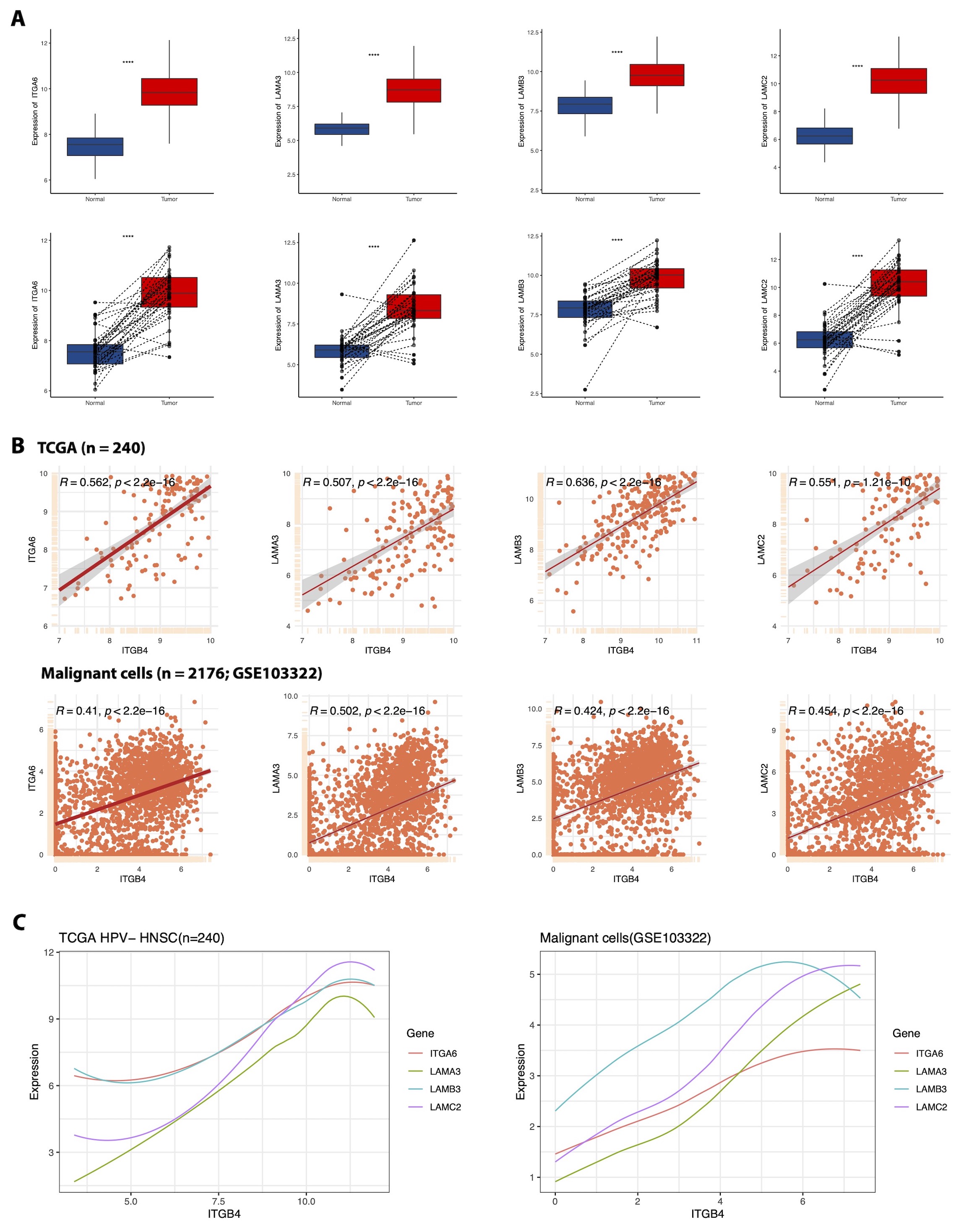
**

**Supplementary Figure 6:** **ITGB4, ITGA6, LAMA3, LAMB3, and LAMC2 expression in HNSCC.** (**A**) ITGB4, ITGA6, LAMA3, LAMB3, and LAMC2 gene expression in normal mucosa (n = 34) and HNSCC (n = 238), and in matched pairs of mucosa and HNSCC (n = 34) from TCGA is shown. (**B**) Scatter plots of ITGB4 correlations with ITGA6, LAMA3, LAMB3, and LAMC2 in HNSCC (n = 240) of TCGA and in n = 2176 malignant single cells from GSE103322 are shown with Spearman correlation and p-value. (**C**) ITGA6, LAMA3, LAMB3, and LAMC2 expression was plotted against ITGB4 with locally weighted smoothing using LOESS in HNSCC of the TCGA cohort (n = 240) and in single malignant HNSCC cells from GSE103322.

**Supplementary Figure 7:** **ITGB4 expression in malignant and non-malignant single cells in different cancer entities.** scRNAseq datasets (n = 79) encompassing were used to screen for ITGB4 expression. (**A**) ITGB4 normalized gene expression (log/transcripts per million/10+1) is depicted in malignant cells, immune cells, and stromal cells from different cancer entities (n = 28) within TISCH for all cancer entities with an expression value >0.5 log (TPM/10+1) in a heatmap with corresponding dataset GSE numbers. (**B**) ITGB4 expression in HNSCC patients (GSE103322) in the indicated cell subsets. (**C**) Violin plot representation of ITGB4 gene expression in malignant cells, immune cells, and stromal cells of HNSCC, pancreatic adenocarcinoma (PAAD), and colorectal carcinoma (CRC).

**Supplementary Figure 8:** **ITGA6 expression in malignant and non-malignant single cells in different cancer entities.** scRNAseq datasets (n = 79) encompassing were used to screen for ITGA36 expression. (**A**) ITGA normalized gene expression (log/transcripts per million/10+1) is depicted in malignant cells, immune cells, and stromal cells from colorectal carcinoma (CRC), HNSCC, and pancreatic adenocarcinoma (PAAD) within TISCH in a heatmap with corresponding dataset GSE numbers. (**B**) ITGA expression in HNSCC patients (GSE103322) in the indicated cell subsets. (**C**) Violin plot representation of ITGA6 gene expression in malignant cells, immune cells, and stromal cells of HNSCC, pancreatic adenocarcinoma (PAAD), and colorectal carcinoma (CRC).

**Supplementary Figure 9:** **LAMA3 expression in malignant and non-malignant single cells in different cancer entities.** scRNAseq datasets (n = 79) encompassing were used to screen for LAMA3 expression. (**A**) LAMA3 normalized gene expression (log/transcripts per million/10+1) is depicted in malignant cells, immune cells, and stromal cells from colorectal carcinoma (CRC), HNSCC, and pancreatic adenocarcinoma (PAAD) within TISCH in a heatmap with corresponding dataset GSE numbers. (**B**) LAMA3 expression in HNSCC patients (GSE103322) in the indicated cell subsets. (**C**) Violin plot representation of LAMA3 gene expression in malignant cells, immune cells, and stromal cells of HNSCC, pancreatic adenocarcinoma (PAAD), and colorectal carcinoma (CRC).

**Supplementary Figure 10:** **LAMB3 expression in malignant and non-malignant single cells in different cancer entities.** scRNAseq datasets (n = 79) encompassing were used to screen for LAMB3 expression. (**A**) LAMB3 normalized gene expression (log/transcripts per million/10+1) is depicted in malignant cells, immune cells, and stromal cells from colorectal carcinoma (CRC), HNSCC, and pancreatic adenocarcinoma (PAAD) within TISCH in a heatmap with corresponding dataset GSE numbers. (**B**) LAMB3 expression in HNSCC patients (GSE103322) in the indicated cell subsets. (**C**) Violin plot representation of LAMB3 gene expression in malignant cells, immune cells, and stromal cells of HNSCC, pancreatic adenocarcinoma (PAAD), and colorectal carcinoma (CRC).

**Supplementary Figure 11:** **LAMC2 expression in malignant and non-malignant single cells in different cancer entities.** scRNAseq datasets (n = 79) encompassing were used to screen for LAMC2 expression. (**A**) LAMC2 normalized gene expression (log/transcripts per million/10+1) is depicted in malignant cells, immune cells, and stromal cells from colorectal carcinoma (CRC), HNSCC, and pancreatic adenocarcinoma (PAAD) within TISCH in a heatmap with corresponding dataset GSE numbers. (**B**) LAMC2 expression in HNSCC patients (GSE103322) in the indicated cell subsets. (**C**) Violin plot representation of LAMC2 gene expression in malignant cells, immune cells, and stromal cells of HNSCC, pancreatic adenocarcinoma (PAAD), and colorectal carcinoma (CRC).

**
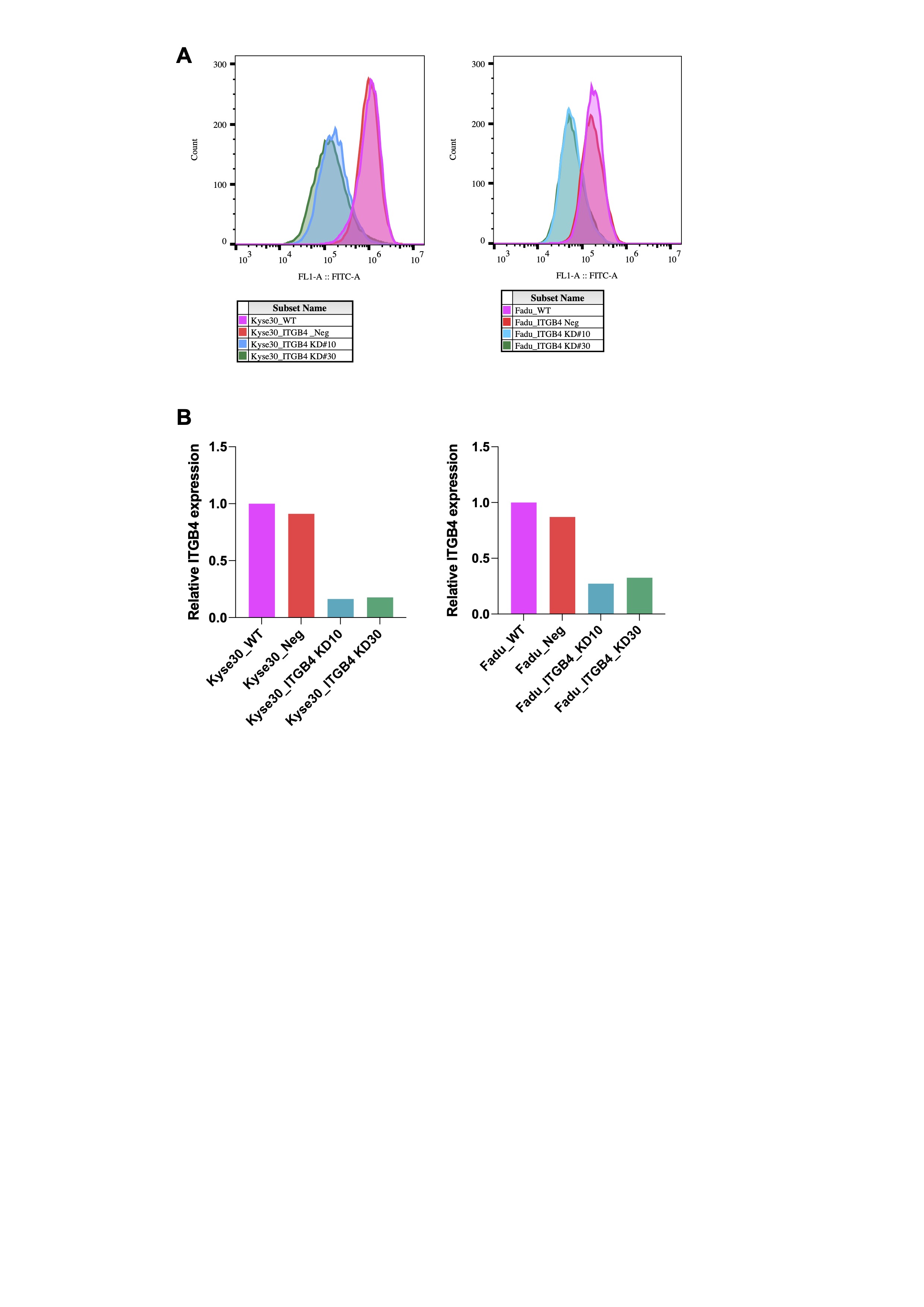
**

**Supplementary Figure 12:** **ITGB4 expression in knockdown clones of Kyse30 and FaDu cells.** (**A**) Wildtype Kyse30 and FaDu cells, control (ITGB4_Neg) and ITGB4-KD clones (ITGB4 KD#10 and #30) were analyzed by flow cytometry for ITGB4 expression. Shown are representative histograms. (**B**) ITGB4 expression was quantified as mean fluorescence intensity and was normalized for the expression in wildtype cells.

**
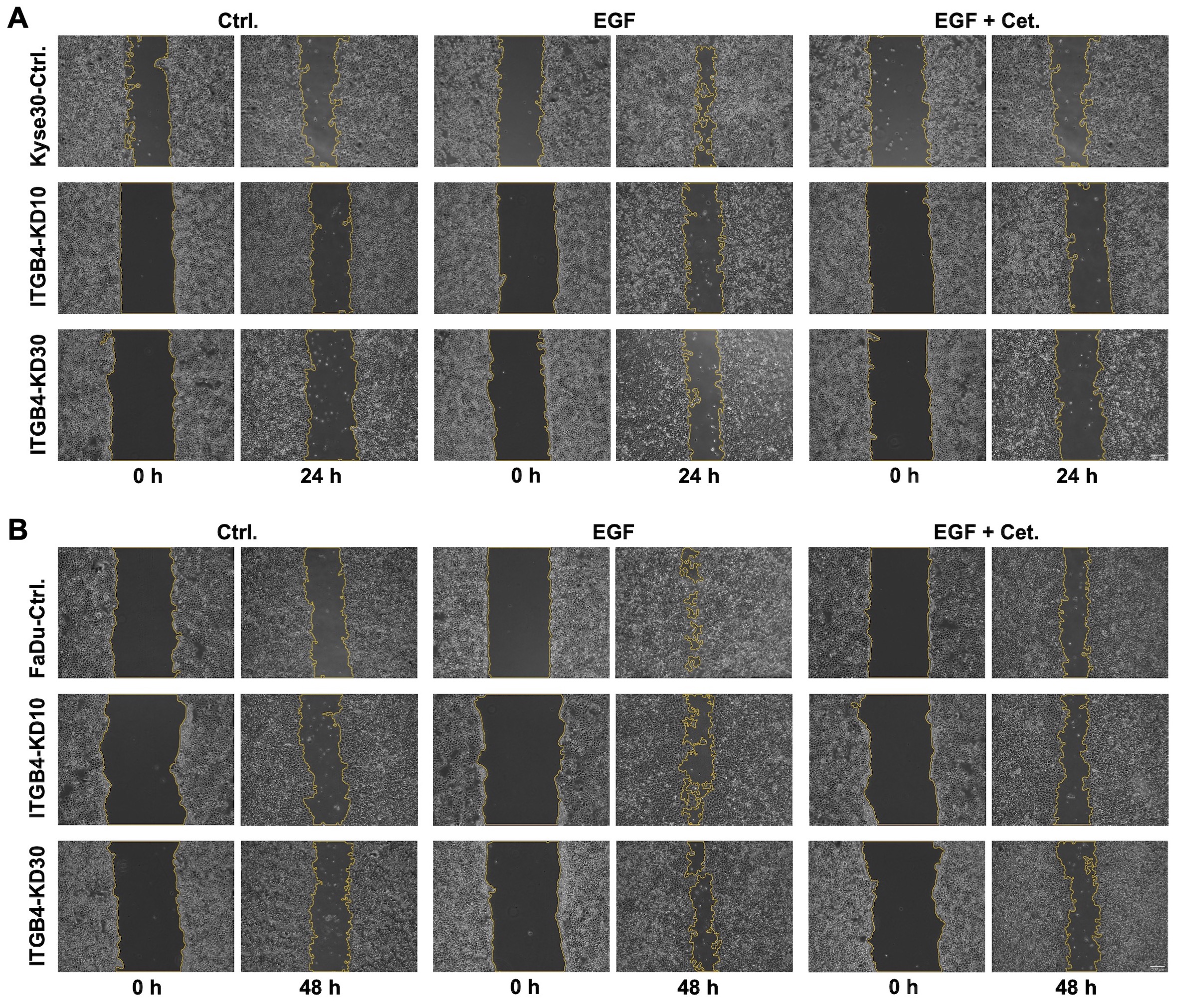
**

**Supplementary Figure 13:** **Wound healing capacity of control and ITGB4-knockdown cell lines.** Kyse30 (**A**) and FaDu cells (**B**) were stably transduced with lentiviruses expressing ITGB4-specific shRNA (ITGB4_KD10 and ITGB4_KD30 represent two different MOI of the virus) or a control shRNA (Neg). Control and ITGB4-knockdown clones were analyzed in a wound healing assay. Shown are representative microscopic images of scratches at 0h and 24h (Kyse30), and at 0h and 48h (FaDu) from n = 3 independent experiments. Scalebars represent 100 µm.


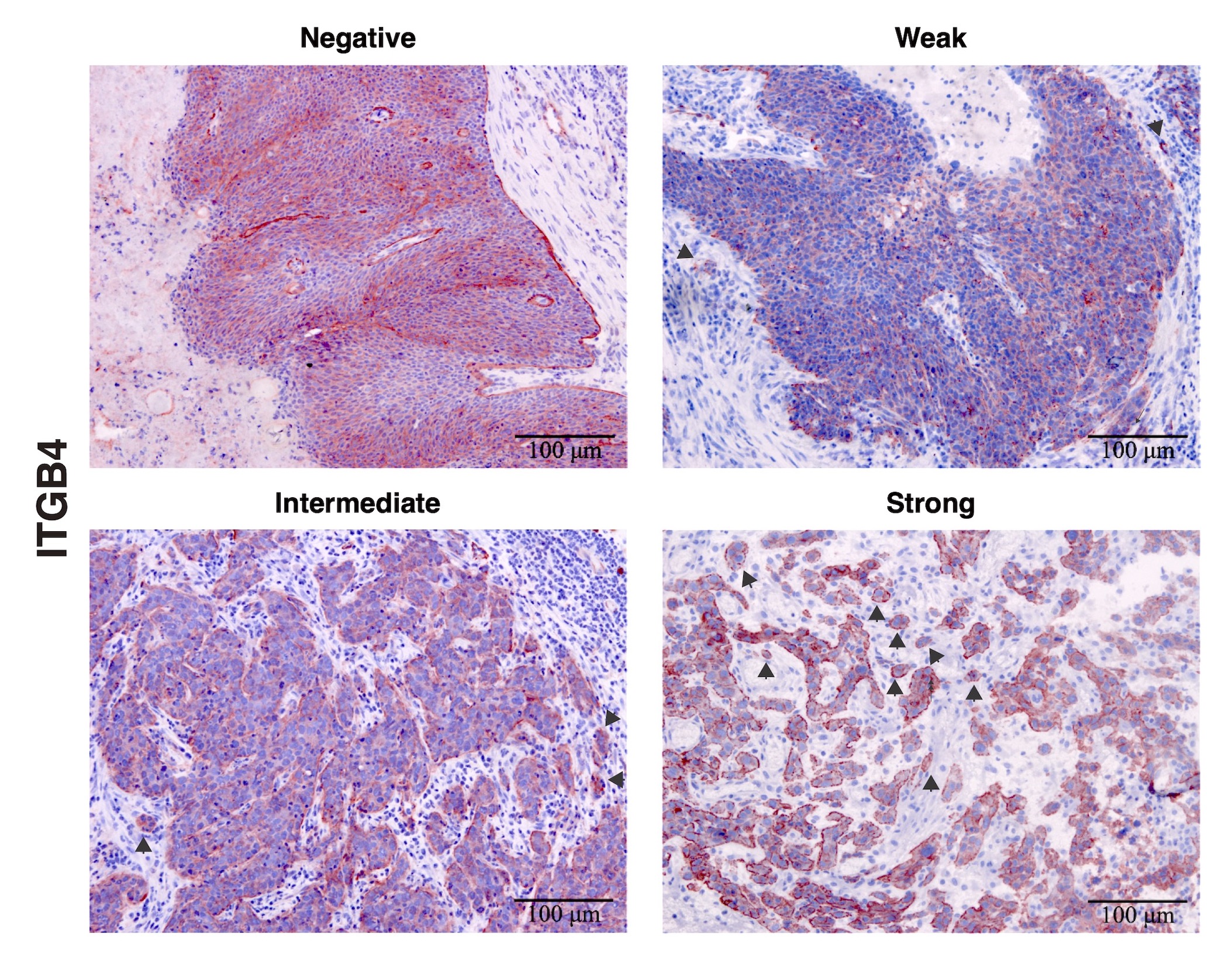


**Supplementary Figure 14:** **Tumor budding intensities in HNSCC.** Examples of budding intensities are shown in ITGB4-stained HNSCC ranging from negative, weak, intermediate to strong. Specimens are stained for ITGB4 expression in red. Arrow heads indicate tumor budding. Scalebars represent 100 µm.
