# Supplementary material for: A transcriptomic map of EGFR-induced epithelial-to-mesenchymal transition identifies prognostic and therapeutic targets for head and neck cancer
Source: Mol Cancer. 2022 Sep 8;21:178. doi: 10.1186/s12943-022-01646-1 (PMC9454230; doi:10.1186/s12943-022-01646-1)
Supplement: Supplementary file 3 — Additional file 3: Supplementary Table 2. TCGA HPV- HNSC cohort. [file 12943_2022_1646_MOESM3_ESM.docx]

TCGA HPV- HNSC cohort

|  | Dead **(N=126)** | Alive **(N=114)** | **Overall** **(N=240)** |
| --- | --- | --- | --- |
| Age |  |  |  |
| Mean (SD) | 64.0 (12.8) | 59.4 (11.8) | 61.8 (12.5) |
| Median [Min, Max] | 64.0 [29.0, 90.0] | 61.0 [19.0, 83.0] | 62.0 [19.0, 90.0] |
| Gender |  |  |  |
| Female | 43 (34.1%) | 29 (25.4%) | 72 (30.0%) |
| Male | 83 (65.9%) | 85 (74.6%) | 168 (70.0%) |
| Primary site |  |  |  |
| Hypopharynx | 1 (0.8%) | 0 (0%) | 1 (0.4%) |
| Larynx | 38 (30.2%) | 32 (28.1%) | 70 (29.2%) |
| Oral Cavity | 80 (63.5%) | 78 (68.4%) | 158 (65.8%) |
| Oropharynx | 7 (5.6%) | 4 (3.5%) | 11 (4.6%) |
| T stage |  |  |  |
| T1 | 6 (4.8%) | 6 (5.3%) | 12 (5.0%) |
| T2 | 34 (27.0%) | 33 (28.9%) | 67 (27.9%) |
| T3 | 42 (33.3%) | 34 (29.8%) | 76 (31.7%) |
| T4a | 43 (34.1%) | 41 (36.0%) | 84 (35.0%) |
| T4b | 1 (0.8%) | 0 (0%) | 1 (0.4%) |
| N stage |  |  |  |
| N0 | 61 (48.4%) | 63 (55.3%) | 124 (51.7%) |
| N1 | 22 (17.5%) | 24 (21.1%) | 46 (19.2%) |
| N2 | 9 (7.1%) | 3 (2.6%) | 12 (5.0%) |
| N2a | 4 (3.2%) | 1 (0.9%) | 5 (2.1%) |
| N2b | 13 (10.3%) | 15 (13.2%) | 28 (11.7%) |
| N2c | 14 (11.1%) | 7 (6.1%) | 21 (8.8%) |
| N3 | 3 (2.4%) | 1 (0.9%) | 4 (1.7%) |
| M stage |  |  |  |
| M0 | 124 (98.4%) | 114 (100%) | 238 (99.2%) |
| M1 | 2 (1.6%) | 0 (0%) | 2 (0.8%) |
| Clinical stage |  |  |  |
| I | 4 (3.2%) | 5 (4.4%) | 9 (3.8%) |
| II | 25 (19.8%) | 24 (21.1%) | 49 (20.4%) |
| III | 29 (23.0%) | 33 (28.9%) | 62 (25.8%) |
| IVA | 62 (49.2%) | 51 (44.7%) | 113 (47.1%) |
| IVB | 4 (3.2%) | 1 (0.9%) | 5 (2.1%) |
| IVC | 2 (1.6%) | 0 (0%) | 2 (0.8%) |
| Follow_up |  |  |  |
| Mean (SD) | 22.2 (30.3) | 32.7 (31.0) | 27.2 (31.0) |
| Median [Min, Max] | 13.0 [0, 211] | 24.4 [0, 142] | 16.4 [0, 211] |

MDACC cohort (GSE42743)

|  | Alive (N=32) | Dead (N=42) | Overall (N=74) |
| --- | --- | --- | --- |
| Age |  |  |  |
| Mean (SD) | 57.6 (13.0) | 58.5 (14.2) | 58.1 (13.6) |
| Median [Min, Max] | 58.5 [28.0, 82.0] | 61.5 [22.0, 84.0] | 59.5 [22.0, 84.0] |
| Gender |  |  |  |
| Female | 5 (15.6%) | 11 (26.2%) | 16 (21.6%) |
| Male | 27 (84.4%) | 31 (73.8%) | 58 (78.4%) |
| SmokStat |  |  |  |
| Current | 12 (37.5%) | 17 (40.5%) | 29 (39.2%) |
| Former | 14 (43.8%) | 16 (38.1%) | 30 (40.5%) |
| NeverSmoker | 6 (18.8%) | 9 (21.4%) | 15 (20.3%) |
| PrimSite |  |  |  |
| Oral cavity | 31 (96.9%) | 40 (95.2%) | 71 (95.9%) |
| Oropharynx | 1 (3.1%) | 2 (4.8%) | 3 (4.1%) |
| Stage |  |  |  |
| I | 3 (9.4%) | 0 (0%) | 3 (4.1%) |
| II | 10 (31.3%) | 6 (14.3%) | 16 (21.6%) |
| III | 8 (25.0%) | 7 (16.7%) | 15 (20.3%) |
| IV | 11 (34.4%) | 29 (69.0%) | 40 (54.1%) |
| Follow-up |  |  |  |
| Mean (SD) | 30.3 (19.8) | 12.5 (14.3) | 20.2 (19.0) |
| Median [Min, Max] | 26.9 [0.230, 92.4] | 7.62 [0.328, 71.9] | 14.5 [0.230, 92.4] |

FHCRC cohort (GSE41613)

|  | Alive (N=46) | Dead (N=51) | Overall (N=97) |
| --- | --- | --- | --- |
| Age |  |  |  |
| 19-39 | 3 (6.5%) | 3 (5.9%) | 6 (6.2%) |
| 40-49 | 8 (17.4%) | 8 (15.7%) | 16 (16.5%) |
| 50-59 | 9 (19.6%) | 19 (37.3%) | 28 (28.9%) |
| 60-88 | 26 (56.5%) | 21 (41.2%) | 47 (48.5%) |
| Gender |  |  |  |
| Female | 15 (32.6%) | 16 (31.4%) | 31 (32.0%) |
| Male | 31 (67.4%) | 35 (68.6%) | 66 (68.0%) |
| Stage |  |  |  |
| I/II | 30 (65.2%) | 11 (21.6%) | 41 (42.3%) |
| III/IV | 16 (34.8%) | 40 (78.4%) | 56 (57.7%) |
| Follow-up |  |  |  |
| Mean (SD) | 66.6 (8.92) | 23.9 (19.9) | 44.1 (26.5) |
| Median [Min, Max] | 65.3 [52.6, 85.0] | 18.4 [0.460, 78.3] | 54.4 [0.460, 85.0] |

LMU cohort

|  | **Alive (N=101)** | **Dead (N=4)** | **Overall (N=105)** |
| --- | --- | --- | --- |
| **Age** |  |  |  |
| Mean (SD) | 62.8 (9.28) | 69.5 (10.4) | 63.0 (9.36) |
| Median [Min, Max] | 63.7 [41.0, 86.4] | 70.0 [59.1, 79.0] | 63.7 [41.0, 86.4] |
| **Gender** |  |  |  |
| Female | 23 (22.8%) | 0 (0%) | 23 (21.9%) |
| Male | 78 (77.2%) | 4 (100%) | 82 (78.1%) |
| **Primary.site** |  |  |  |
| Larynx/hypopharynx | 35 (34.7%) | 2 (50.0%) | 37 (35.2%) |
| Oral cavity | 19 (18.8%) | 2 (50.0%) | 21 (20.0%) |
| Oropharynx | 47 (46.5%) | 0 (0%) | 47 (44.8%) |
| **Tstage** |  |  |  |
| T1 | 10 (9.9%) | 1 (25.0%) | 11 (10.5%) |
| T2 | 36 (35.6%) | 2 (50.0%) | 38 (36.2%) |
| T3 | 32 (31.7%) | 1 (25.0%) | 33 (31.4%) |
| T4a | 19 (18.8%) | 0 (0%) | 19 (18.1%) |
| Unknown | 4 (2.0%) | 0 (0%) | 4 (3.8%) |
| **Nstatus** |  |  |  |
| N- | 35 (34.7%) | 4 (100%) | 39 (37.1%) |
| N+ | 63 (62.4%) | 0 (0%) | 63 (60.0%) |
| Unknown | 3 (3.0%) | 0 (0%) | 3 (2.9%) |
| **OS_followup** |  |  |  |
| Mean (SD) | 16.2 (11.6) | 12.8 (9.40) | 16.1 (11.5) |
| Median [Min, Max] | 14.6 [0.0300, 42.1] | 13.5 [1.12, 23.1] | 14.6 [0.0300, 42.1] |

IRCCS cohort (GSE65021)

|  | Long PFS (N=14) | Short PFS (N=26) | Overall (N=40) |
| --- | --- | --- | --- |
| Age |  |  |  |
| Mean (SD) | 62.7 (7.62) | 57.3 (10.5) | 59.2 (9.84) |
| Median [Min, Max] | 62.5 [45.0, 75.0] | 61.0 [36.0, 73.0] | 62.0 [36.0, 75.0] |
| Gender |  |  |  |
| Male | 11 (78.6%) | 20 (76.9%) | 31 (77.5%) |
| Female | 3 (21.4%) | 6 (23.1%) | 9 (22.5%) |
| Grade |  |  |  |
| I | 1 (7.1%) | 1 (3.8%) | 2 (5.0%) |
| II | 7 (50.0%) | 13 (50.0%) | 20 (50.0%) |
| III | 6 (42.9%) | 12 (46.2%) | 18 (45.0%) |
| Tstage |  |  |  |
| I | 1 (7.1%) | 2 (7.7%) | 3 (7.5%) |
| II | 2 (14.3%) | 6 (23.1%) | 8 (20.0%) |
| III | 5 (35.7%) | 4 (15.4%) | 9 (22.5%) |
| VI | 6 (42.9%) | 14 (53.8%) | 20 (50.0%) |
