# Supplementary material for: A transcriptomic map of EGFR-induced epithelial-to-mesenchymal transition identifies prognostic and therapeutic targets for head and neck cancer
Source: Mol Cancer. 2022 Sep 8;21:178. doi: 10.1186/s12943-022-01646-1 (PMC9454230; doi:10.1186/s12943-022-01646-1)
Supplement: Supplementary file 4 — Additional file 4: Supplementary Table 3. Gene expression correlation with ITGB4 in the HPV-negativeTCGA cohort. Batch correlation analysis identified correlations of geneexpression with integrin beta 4 (ITGB4). Gene ID, Spearman correlation, andp-value are indicated for the top ten positively (co-regulated) and negativelycorrelated genes (counterregulated). [file 12943_2022_1646_MOESM4_ESM.docx]

**Supplementary Table 3**: Gene expression correlation with ITGB4 in the HPV-negative TCGA cohort. Batch correlation analysis identified correlations of gene expression with integrin beta 4 (ITGB4). Gene ID, Spearman correlation, and p-value are indicated for the top ten positively (co-regulated) and negatively correlated genes (counterregulated).

| **Co-regulated** | **Gene ID** | **Correlation (Spearman)** | **p-value** |
| --- | --- | --- | --- |
| #1 | ITGA3 | 0.77 | 2.47e-48 |
| #2 | TNS4 | 0.733 | 8.36e-41 |
| #3 | PXN | 0.71 | 1.11e-37 |
| #4 | GJB3 | 0.67 | 2.44e-33 |
| #5 | LAMC2 | 0.66 | 3.92e-32 |
| #6 | CARD10 | 0.66 | 5.06e-32 |
| #7 | COL17A1 | 0.66 | 2.10e-31 |
| #8 | LAMA3 | 0.63 | 3.04e-28 |
| #9 | LAMB3 | 0.62 | 3.23e-27 |
| #10 | VAV2 | 0.60 | 2.03e-25 |
| **Counter-regulated** | **Gene ID** | **Correlation (Spearman)** | **p-value** |
| #1 | CREG1 | -0.47 | 4.55e-15 |
| #2 | CA5B | -0.45 | 1.27e-13 |
| #3 | REPIN1 | -0.44 | 3.27e-13 |
| #4 | GULP1 | -0.44 | 7.16e-13 |
| #5 | AFF3 | -0.44 | 8.50e-13 |
| #6 | METTL9 | -0.43 | 1.63e-12 |
| #7 | WDSUB1 | -0.43 | 2.13e-12 |
| #8 | C12ORF57 | -0.43 | 2.36e-12 |
| #9 | SUSD4 | -0.43 | 2.37e-12 |
| #10 | EID3 | -0.43 | 3.03e-12 |
